# Low-Dose Lithium for Mild Cognitive Impairment: A Pilot Randomized Clinical Trial

**DOI:** 10.1001/jamaneurol.2026.0072

**Published:** 2026-03-02

**Authors:** Ariel G. Gildengers, Tamer S. Ibrahim, Stewart J. Anderson, James E. Emanuel, Tales Santini, Jihui L. Diaz, Brian J. Lopresti, Sarah K. Royse, Oscar L. Lopez, Xuemei Zeng, Bruno de Almeida, Salem K. Alkhateeb, Cong Chu, Thomas K. Karikari, Laisze Lee, Andrea M. Weinstein, Meryl A. Butters

**Affiliations:** 1 Department of Psychiatry, University of Pittsburgh School of Medicine, Pittsburgh, Pennsylvania; 2 Department of Bioengineering, University of Pittsburgh, Pittsburgh, Pennsylvania; 3 Department of Biostatistics, University of Pittsburgh School of Public Health, Pittsburgh, Pennsylvania; 4 School of Nursing, Duke University, Durham, North Carolina; 5 Department of Radiology, University of Pittsburgh School of Medicine, Pittsburgh, Pennsylvania; 6 Department of Neurology, University of Pittsburgh School of Medicine, Pittsburgh, Pennsylvania

## Abstract

**Importance:**

Lithium deficiency may contribute to Alzheimer disease pathogenesis. No randomized clinical trial has examined lithium’s effects on cognition, neuroimaging, and plasma biomarkers in mild cognitive impairment (MCI).

**Objective:**

To examine the feasibility, safety, and preliminary efficacy of lithium carbonate for delaying cognitive decline in older adults with MCI.

**Design, Setting, and Participants:**

This single-site, randomized, double-blind, placebo-controlled pilot feasibility clinical trial was conducted at the University of Pittsburgh School of Medicine from February 2018 to August 2024, with 2-year follow-up. Analyses used linear mixed-effects models in the intention-to-treat population. Adults aged 60 years or older with MCI who were free of major psychiatric or neurologic illness and contraindications to lithium were included. Of 170 individuals assessed, 83 were randomized (41 lithium vs 42 placebo), with 80 starting treatment (41 lithium vs 39 placebo). Data were analyzed from August 2024 to December 2025.

**Intervention:**

Daily low-dose lithium carbonate or placebo for 2 years.

**Main Outcomes and Measures:**

Six prespecified coprimary outcomes included cognitive performance (California Verbal Learning Test-II [CVLT-II] delayed recall, Brief Visuospatial Memory Test-Revised, preclinical Alzheimer cognitive composite), hippocampal volume, cortical gray matter volume, and brain-derived neurotrophic factor.

**Results:**

Among 80 participants (mean [SD] age, lithium: 72.93 [8.77] years; placebo: 71.22 [6.47] years; 56% female), none of the 6 coprimary outcomes met the prespecified significance threshold. Mean (SD) CVLT-II baseline scores were 7.95 (3.4) for lithium and 7.90 (3.9) for placebo; scores declined 1.42 points annually in the placebo group vs 0.73 points in the lithium group (difference, 0.69 points per year; 95% CI, 0.01-1.37; *P* = .05). Hippocampal and cortical volumes showed a decline over time in both groups, but no significant treatment × time interactions. Serious adverse events occurred in 12 of 41 (29%) receiving lithium vs 9 of 39 (23%) receiving placebo; none were definitely treatment related. One death occurred in the placebo group. Common adverse events included increased creatinine levels (12 of 41 [29%] with lithium vs 12 of 39 [31%] with placebo), diarrhea (12 of 41 [29%] vs 6 of 39 [15%]), tiredness (12 of 41 [29%] vs 6 of 39 [15%]), and tremor occurrence (10 of 41 [24%] vs 6 of 39 [15%]).

**Conclusions and Relevance:**

This pilot randomized clinical trial established feasibility, confirmed safety and tolerability, and generated effect size estimates for future trials of low-dose lithium in MCI. None of the coprimary outcomes met the prespecified significance threshold.

**Trial Registration:**

ClinicalTrials.gov Identifier: NCT03185208

## Key Points

**Question:**

Can low-dose lithium treatment delay cognitive decline in older adults with mild cognitive impairment?

**Findings:**

In this pilot randomized clinical trial of 80 participants, none of the 6 coprimary outcomes reached the prespecified significance threshold; for verbal memory, scores declined by 1.42 points annually in the placebo group vs 0.73 points in the lithium group, which did not meet the prespecified threshold for multiple comparisons. Exploratory analyses suggested possible larger effects among amyloid-positive participants.

**Meaning:**

This pilot trial provides effect size estimates and methodological insights to inform adequately powered confirmatory trials of low-dose lithium in older adults with amyloid-positive mild cognitive impairment.

## Teaser

This pilot randomized clinical trial evaluates the feasibility, safety, and methodological considerations of low-dose lithium treatment in older adults with mild cognitive impairment.

## Introduction

Lithium deficiency, resulting from its sequestration by amyloid plaques, may underlie the multisystem neurodegeneration of Alzheimer disease (AD).^1^ This finding provides a framework for understanding evidence that lithium can protect against dementia in studies ranging from cellular and animal experiments to human clinical trials to epidemiological investigations.^2,3,4,5,6,7,8,9,10,11,12^ A 2015 systematic review and meta-analysis of the 3 randomized clinical trials (RCTs) available at that time, aggregating 232 participants with AD and mild cognitive impairment (MCI), found that lithium significantly decreased cognitive decline compared with placebo.^13^ While not all studies have shown that lithium is neuroprotective, the evidence suggests that lithium deficiency may represent a modifiable risk factor for AD. A key mechanism underlying lithium’s neuroprotection appears to be its inhibition of GSK-3α/β.^1,14^ Lithium also increases brain-derived neurotrophic factor (BDNF) expression and activity, which may additionally contribute to neuroprotection.^14^

To our knowledge, no study has examined lithium’s human effects in a prospective RCT that combines cognitive assessment with neuroimaging and plasma biomarkers. The methods used in the Lithium as a Treatment to Prevent Impairment of Cognition in Elders (LATTICE) pilot-feasibility RCT have been reported.^15^ Here, we report the main outcomes of the study examining the potential disease-modifying properties of lithium in individuals with MCI in delaying conversion to dementia. The study addressed the following hypotheses: (1) participants randomized to take lithium for 2 years, compared with placebo, will better maintain cognitive function, primarily in memory, which will be associated with changes in GSK-3β activity and BDNF levels; and (2) participants randomized to take lithium for 2 years, compared with placebo, will have larger hippocampal volumes (ie, lower rate of reduction) and lower total gray matter thinning, which will be associated with changes in GSK-3β activity and BDNF levels and better cognitive function, primarily in memory.

## Methods

### Study Design

LATTICE was a single-site, randomized clinical trial conducted at the University of Pittsburgh School of Medicine. The University of Pittsburgh Human Research Protection Office approved the protocol (Supplement 1). The study was overseen by an independent Data Safety and Monitoring Board (DSMB) that reviewed and approved study procedures before enrollment began on September 1, 2017. The DSMB met approximately every 6 months postenrollment and approved all proposed study modifications and requests for study continuation. Protocol modifications made during trial conduct are detailed in our published methodology report.^15^ There was no formal patient or public involvement in the design, conduct, or reporting of this research. All participants provided written informed consent. This study was conducted and reported according to CONSORT guidelines.

### Participants

Eligibility required (1) age 60 years or older; (2) diagnosis of MCI per Petersen criteria, operationalized as cognitive performance 1 to 2 SDs below the age-adjusted and education-adjusted norms in at least 1 cognitive domain; (3) preserved activities of daily living; (4) absence of major psychiatric illness per Mini-International Neuropsychiatric Interview^16^ (MINI) structured interview; (5) absence of major neurologic illness; (6) no contraindications to lithium; and (7) ability to complete neuropsychological testing (excluding those with nonremediable sensory or motor impairments, such as blindness). Race and ethnicity were self-reported by participants using categories defined by the National Institutes of Health (NIH). Race categories included Asian, Black or African American, and White. Ethnicity was reported separately as Hispanic or Latino or not Hispanic or Latino. Race and ethnicity were assessed to characterize the study sample and to meet NIH requirements for reporting demographic characteristics in clinical research.

### Recruitment and Screening Methods

Recruitment involved senior center presentations, educational outreach, internet advertising, University of Pittsburgh Alzheimer’s Disease Research Center partnerships, and primary care collaborations. The occurrence of the COVID-19 pandemic necessitated transitioning from in-person to digital and print recruitment methods. Initial screening helped identify candidates for comprehensive evaluation by excluding potential participants who were clearly cognitively normal or severely cognitively impaired.

Before the COVID-19 pandemic, the team conducted screening in person using 3 assessments: Modified Mini-Mental State Examination (3MS),^17^ Trail Making Test Parts A and B (TMT A/B),^18^ and Quick Mild Cognitive Impairment screen (Qmci).^19^ Participants qualified for a comprehensive evaluation if they scored beyond 1 SD below expected performance on any single test. Exclusion criteria were performance exceeding 2 SDs below expected on 2 or more tests, or 3MS scores less than 84. The Qmci was age adjusted and education adjusted, while TMT norms incorporated age, education, sex, and race factors.

During the COVID-19 pandemic, screening shifted to telephone assessments using the modified Telephone Interview for Cognitive Status (mTICS)^20^ and Hayling Sentence Completion Test (HSCT).^21^ Participants qualified for comprehensive evaluation with mTICS scores in the MCI range (19-38 of 50) or HSCT scaled scores of 4 or less on any component.

Those who qualified for comprehensive evaluation underwent in-person assessment with the MINI to exclude major psychiatric illness and the medication management portion of the Performance Assessment of Self-Care Skills (PASS)^22^ to confirm safe medication handling ability. Eligible participants then provided written informed consent in accordance with the Helsinki protocol and completed a comprehensive neuropsychological evaluation.

Participants underwent comprehensive neuropsychological evaluation, including Clinical Dementia Rating (CDR)^23^; Everyday Cognition Scale (ECog),^24^ with both self and informant reports; the Wide Range Achievement Test-4th edition (WRAT-4)^25^ Reading subtest; Boston Naming Test^26^; Clock Drawing Test^27^; the Wechsler Adult Intelligence Scale (WAIS)-IV Digit Span subtest^28^; the Repeatable Battery for the Assessment of Neuropsychological Status (RBANS),^29^ using 2 counterbalanced alternate forms to minimize practice effects; and select Delis-Kaplan Executive Function System (D-KEFS)^30^ subtests (Verbal Fluency, Trail Making Test, and Color Word Interference). Functional assessment used the PASS subtests of shopping, medication management, and bill payment.^31^ Trained psychometrists administered the battery of assessments, lasting 4 to 5 hours with breaks. If the participant entered the RCT, these tests were repeated at 1 and 2 years, excluding the WRAT-4 Reading, to track cognitive status and determine whether participants reverted to normal cognition, remained stable with MCI, or progressed to dementia.

MCI diagnosis required multidisciplinary adjudication (neuropsychologist, neurologist, geriatric psychiatrist) using National Alzheimer’s Coordinating Center/Revised Petersen criteria: (1) subjective cognitive concern (Ecog), (2) objective impairment 1 to 2 SDs below expectation on 2 tests within 1 domain or 3 tests across domains, (3) preserved functional independence (CDR and PASS), and (4) absence of dementia.^32,33^ Performances were standardized using demographic-adjusted norms. Participants who met all inclusion criteria, had no exclusion criteria, and agreed to enter the RCT received PET imaging to measure brain amyloid. Participants received modest stipends for completing study assessments.

### Randomization and Masking

Participants were randomly assigned (1:1) to lithium or placebo using permuted blocks with even-numbered block sizes, ranging from 2 to 16. Randomization was stratified by the presence of amyloid-beta (Aβ) plaque (positive, negative, or unknown). The study statistician (S.J.A.) generated the randomization sequence using the sample function in R statistical software. He had no role in enrollment or treatment assignment. The data manager assigned participants to trial groups and had no direct contact with participants.

### Procedures

Participants who entered the RCT received either a 150-mg or 300-mg dose of lithium carbonate or placebo in identical over-encapsulated pills. Participants started 1 pill daily or every other day, based on general medical status and concomitant medications. Doses were adjusted weekly to the maximum tolerated dose and decreased if needed to achieve tolerability. All participants underwent lithium blood level monitoring. An unmasked team member obtained lithium level results and reported either actual values (lithium group) or algorithm-generated false values (placebo group) to the masked research team, maintaining treatment allocation concealment except during medical emergencies. Participants were seen weekly during initial titration, then roughly every 3 months through study completion. eTable 1 in Supplement 2 displays the assessments.

### Neurocognitive Assessment

The RCT cognitive assessment battery included (1) an adapted preclinical Alzheimer cognitive composite (PACC)^34^ consisting of the 3MS, RBANS delayed list recall, RBANS coding, D-KEFS Trail Making condition 4, and PASS cognitive-instrumental activities of daily living (IADL) tasks; (2) the Brief Visuospatial Memory Test-Revised (BVMT-R)^35^; and (3) the California Verbal Learning Test-II (CVLT-II).^36^ The BVMT-R and CVLT-II were administered after MCI diagnosis was confirmed and before starting the study medication.

### Magnetic Resonance Imaging

Ultrahigh-field 7-T magnetic resonance imaging (MRI) was conducted using an MRI scanner (MAGNETOM; Siemens) with radio frequency coil hardware (Tac G1 and G2/Tic Tac Toe) that delivers whole-brain homogenous imaging at 7T.^37,38^ Longitudinal baseline-weighted images (0.75-mm isotropic resolution) were processed using FreeSurfer, version 8 with a pipeline adapted for 7-T images, including bias and gradient distortion corrections.^39^ Manual quality assurance was performed. Participants or data points with excessive motion (2 participants at all 3 time points: baseline [T1], year 1 [T2], and year 2 [T3]) or incidental findings (1 participant at all 3 time points and 1 participant at time point 2) were excluded. Morphometrics were adjusted for age at baseline, sex, intracranial volume, and follow-up duration.

### Positron Emission Tomography Imaging

For concurrent imaging with positron emission tomography (PET) and MRI, we used a Biograph mMR scanner (Siemens) with simultaneous 3-T capability. PET imaging with [^11^C]-labeled Pittsburgh Compound-B (PiB; 15.0 mCi nominal) was used to assess cerebral Aβ plaque burden (acquired 50 to 70 minutes postinjection), and concurrent T1-weighted magnetization-prepared rapid gradient-echo and Dixon sequences were collected for PET image sampling and attenuation correction. FreeSurfer-based analysis yielded standardized uptake value ratios (SUVRs) for a composite 9-region global index of Aβ burden (GBL9). Aβ positivity (Aβ+) was defined as GBL9 SUVR of 1.346 or greater based on sparse k-means clustering from 61 cognitively normal participants.^40,41^

### Blood Sampling

Safety monitoring included a basic metabolic panel and thyroid-stimulating hormone at T1, T2, and T3, and an electrocardiogram at baseline and follow-up as needed; urinalysis and urine osmolality at baseline and T3; and lithium levels at biweekly titration visits and quarterly visits. Weekly safety laboratory review monitored kidney, thyroid, and parathyroid function. Biomarker blood collection occurred at entry, then every 6 months for apolipoprotein E (APOE) genotype (baseline only), to measure BDNF using a NULISAseq CNS Disease Panel 120 assay (Alamar Biosciences).^42^

### Additional Assessments

Medical comorbidity (Cumulative Illness Rating Scale-Geriatric [CIRS-G]),^43^ cardiovascular risk (Framingham Stroke Risk Profile [FSRP]),^44^ mood (Patient Health Questionnaire-9 [PHQ-9]),^45^ physical activity (Physical Activity Scale for the Elderly [PASE]),^46^ drug compliance (Brief Adherence Rating Scale [BARS]),^47^ and adverse effects (Udvalg for Kliniske Undersøgelser [UKU] Side Effect Rating Scale^48^ and spontaneous reporting of adverse effects [SRSE]) were assessed. Anticholinergic burden was calculated by summing medication scores (0 indicates no activity, 1 indicates serum activity without cognitive effects, 2 indicates clinically relevant activity, 3 indicates high potency).^49^

### Outcomes

Six coprimary outcomes were prespecified: cognitive measures including overall cognitive function (PACC), verbal memory (CVLT-II delayed recall), and visual memory (BVMT-R delayed recall); neuroimaging measures including hippocampal volume and total cerebral cortical gray matter (cortex volume); and 1 plasma biomarker (BDNF). GSK-3 activity was originally planned as an additional biomarker outcome, but the assays failed quality control and could not be assessed. We assessed compliance, safety, and adverse events with the BARS and UKU adverse effect rating scale and SRSE.^15^

As required by the National Institute on Aging, we impaneled an external DSMB with expertise in clinical trials, geriatrics, AD and AD-related dementias, and statistics. Investigators met with the DSMB semiannually. Participants’ clinical status was reviewed weekly, and all serious adverse events were reported to the DSMB within 24 hours.

### Statistical Analysis

The statistical analysis plan was specified previously and approved by the DSMB before enrollment in September 2017 (eMethods in Supplement 2). Data were analyzed from August 2024 to December 2025.

Power calculations based on 80 randomized participants (64 completers with 32 per group) with 3 measurement time points indicated that by using a 2-sided significance level of .05 and 80% power, we could detect a medium effect size (Cohen *d* = 0.57) when observations had a 0.5 correlation across time. We also computed the effect size for a 2-sided significance level of .01, a more conservative alpha for testing multiple outcomes, and a power of 80%, resulting in an effect size that ranged from 0.54 to 0.72 as the correlation between repeated observations ranged from 0.1 to 0.6. We used the .01 threshold for our primary analyses.

Analyses began with descriptive methods and 2-sample tests (*t* tests, Wilcoxon tests, χ^2^ tests) to compare the intervention and control groups. Baseline characteristics and outcome data were compared using *t* tests, rank sum tests, or χ^2^ tests as appropriate. All tests were 2-sided. We report uncorrected *P* values and note which findings meet the *P* < .01 threshold. Primary longitudinal analyses employed linear mixed-effects trajectory models, including time (as a continuous variable using actual sampling times), group, and time-by-group interaction terms. Intercept and slope parameters were assumed to have random effect components. Of primary interest was the treatment × time interaction effect, evaluating whether decline rates differed between groups. Missing longitudinal outcomes were handled under the missing at random assumption using mixed-effects models.

Prespecified completer analyses were performed using simple *t* tests to compare treatment groups according to individuals’ change in outcome values between baseline (time point 1) and study end (time point 3). The completer analyses included a preplanned subgroup analysis of participants who were Aβ+ based on PiB-PET. Statistical analyses were performed using SAS, version 9.4 (SAS Institute Inc) and statistical graphics through R, version 4.4.2 (R Foundation for Statistical Computing).

## Results

**Figure 1.**
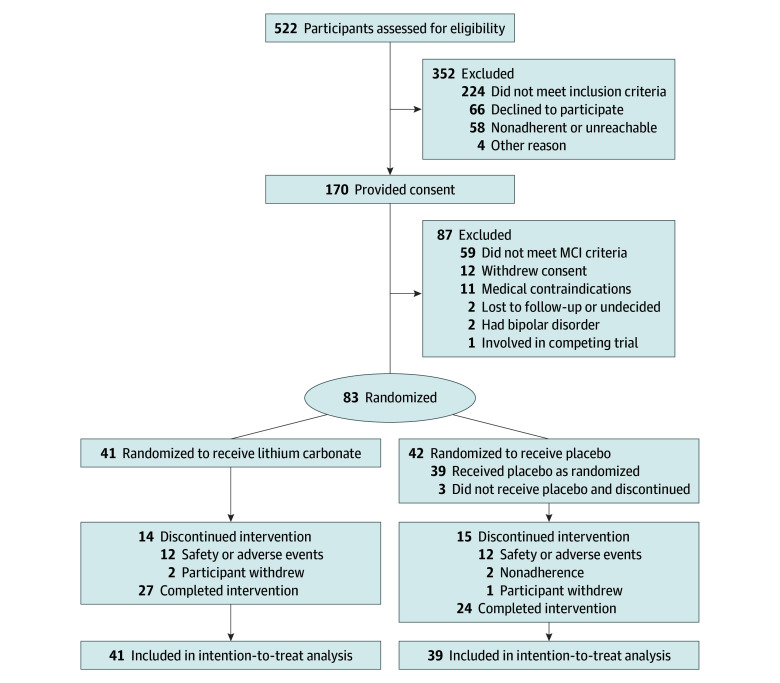
CONSORT Diagram of Participant Flow in the LATTICE Trial Participant flow through screening, randomization, and follow-up phases. Of 170 individuals assessed for eligibility, 83 were randomized (41 lithium vs 42 placebo), with 80 starting treatment (41 lithium vs 39 placebo). CONSORT indicates Consolidated Standards of Reporting Trials; LATTICE, Lithium as a Treatment to Prevent Impairment of Cognition in Elders; MCI, mild cognitive impairment.

Between February 2, 2018, and August 6, 2022, 170 individuals were assessed for eligibility. Of these, 12 withdrew, and 75 met exclusion criteria. Thus, 83 participants were randomized (41 lithium and 42 placebo). Three of the 83 randomized participants withdrew before starting the trial, leaving 80 (41 lithium and 39 placebo) (Figure 1). Among participants in the lithium group, the mean (SD) age was 72.93 (8.77) years, with 23 female (56%) and 18 male (44%); among those in the placebo group, the mean (SD) age was 71.22 (6.47) years, with 22 female (56%) and 17 male (44%). The trial was completed as planned with no early stopping.

**Figure 2.**
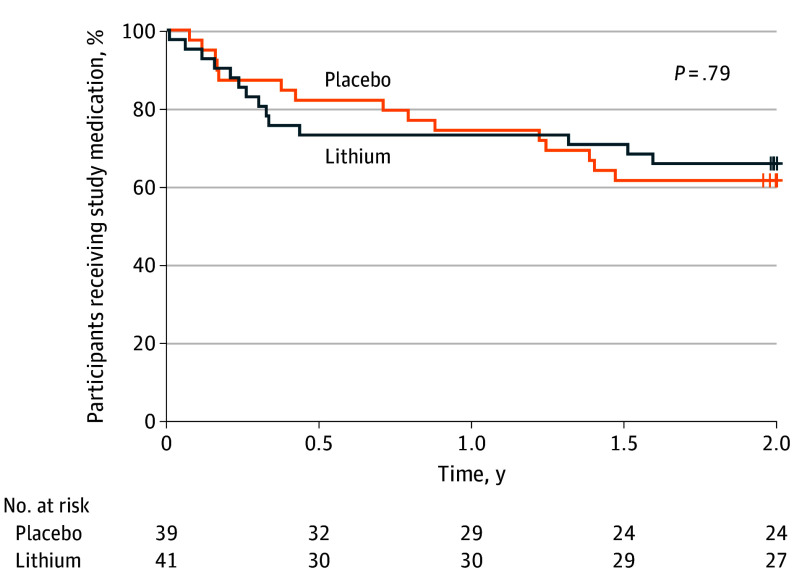
Kaplan-Meier Curve Showing Time to Treatment Discontinuation by Treatment Group Kaplan-Meier curve showing the proportion of participants remaining on assigned treatment (lithium vs placebo) throughout the 2-year study period. Testing for nonproportional hazards between groups: Grambsch-Therneau test, *P* = .29; Tarone-Ware test, *P* > .75; Gehan test, *P* > .75. Numbers at risk shown below x-axis.

Baseline demographic and clinical data were well balanced (Table 1). Study medication discontinuation occurred in 29 of 80 participants (36%) overall: 14 of 41 participants (34%) in the lithium group by 2 years and 15 of 39 (38%) in the placebo group by 2 years (Figure 2). Testing for nonproportional hazards in medication discontinuation between treatment groups using the Grambsch-Therneau test^50^ yielded *P* = .29, and additional tests weighted toward early discontinuation events (Tarone-Ware^51^ and Gehan^52^ tests) both yielded *P* > .75. Retention for outcome assessments exceeded 80% at year 2.

**Table 1.** Baseline Characteristics by Treatment Group^a^

Characteristic	Lithium (n = 41)	Placebo (n = 39)
**Demographic**		
Age, mean (SD), y	72.93 (8.77)	71.22 (6.47)
Sex, No. (%)		
Female	23 (56)	22 (56)
Male	18 (44)	17 (44)
Education, mean (SD), y	15.46 (2.66)	16.54 (1.80)
Race, No. (%)		
Asian	0	1 (3)
Black or African American	3 (7)	5 (13)
White	38 (93)	33 (85)
Ethnicity, No. (%)		
Hispanic or Latino	0	1 (3)
Not Hispanic or Latino	41 (100)	38 (97)
**Clinical characteristics**		
Aβ status, No.		
Aβ−	27 (66)	27 (69)
Aβ+	11 (27)	10 (26)
Aβ unknown	3 (7)	2 (5)
MCI type, No. (%)		
Amnestic	34 (83)	28 (72)
Nonamnestic	7 (17)	11 (28)
APOE ε4 carriers, No. (%)	15 (37)	13 (33)
**Clinical measures, mean (SD)**		
FSRP	0.13 (0.12)	0.11 (0.12)
CIRS-G total	11.07 (4.67)	9.90 (3.61)
Creatinine, mg/dL	0.84 (0.16)	0.92 (0.14)
GFR, mL/min per 1.73 m^2^	84.23 (11.74)	77.95 (13.11)
Medication count	7.78 (5.22)	6.64 (4.84)
Anticholinergic burden	2.53 (2.39)	2.34 (2.85)
PASE	117.01 (73.65)	90.20 (50.91)
PHQ-9	3.88 (3.27)	3.49 (3.46)
**Neuropsychological and neuroimaging characteristics**		
BVMT-R, mean (SD)^b^	6.23 (3.11) [n = 40]	6.56 (2.86) [n = 39]
CVLT-II, mean (SD)^b^	7.95 (3.40) [n = 40]	7.90 (3.90) [n = 39]
PACC score, mean (SD)^b^	−0.36 (3.59) [n = 41]	0.41 (3.16) [n = 36]
Brain volume, mean (SD), mm^3^	n = 33	n = 29
Hippocampal^b^	7387.23 (1293.01)	7199.39 (801.07)
Cerebral cortical gray matter^b^	416 997.07 (51 633.65)	410 702.08 (40 439.38)
Plasma biomarker data	n = 36	n = 37
BDNF (log-transformed), mean (SD)^b^	14.61 (0.83)	14.41 (1.47)

Abbreviations: APOE, apolipoprotein E; Aβ, amyloid-beta; BDNF, brain-derived neurotrophic factor; BVMT-R, Brief Visuospatial Memory Test-Revised (delayed recall); CIRS-G, Cumulative Illness Rating Scale-Geriatric; CVLT-II, California Verbal Learning Test-II (long delayed free recall); FSRP, Framingham Stroke Risk Profile; GFR, glomerular filtration rate; MCI, mild cognitive impairment; PACC, Preclinical Alzheimer Cognitive Composite; PASE, Physical Activity Scale for the Elderly; PHQ-9, Patient Health Questionnaire-9.

^a^ Baseline characteristics are presented descriptively without statistical testing. In randomized clinical trials, any differences between groups at baseline are by definition due to chance, and statistical testing is not appropriate.

^b^ Coprimary outcome measures. One participant in the lithium group did not complete BVMT-R and CVLT-II due to substantial prior familiarity with these tests.

Among participants who completed the study, the mean (SD) daily dose of lithium was 195 (150) mg with a mean (SD) serum level of 0.17 (0.13) mEq/L (maximum, 0.5 mEq/L) and 98% pill compliance. The mean (SD) false dose in the placebo group was 279 (147) mg daily with 97% pill compliance.

There was 1 death in the placebo group that was not study related. There were no deaths in the lithium group. There were 41 serious adverse events (25 lithium vs 16 placebo), with none definitely related to study medication. Common adverse events included increased creatinine, diarrhea, tiredness, and tremor (eTables 2 and 3 in Supplement 2). At study end, the percentages of participants and staff who correctly guessed treatment assignment were as follows: 56% of participants, 55% of neuropsychology staff, 47% of clinical staff, and 54% of investigators.

**Figure 3.**
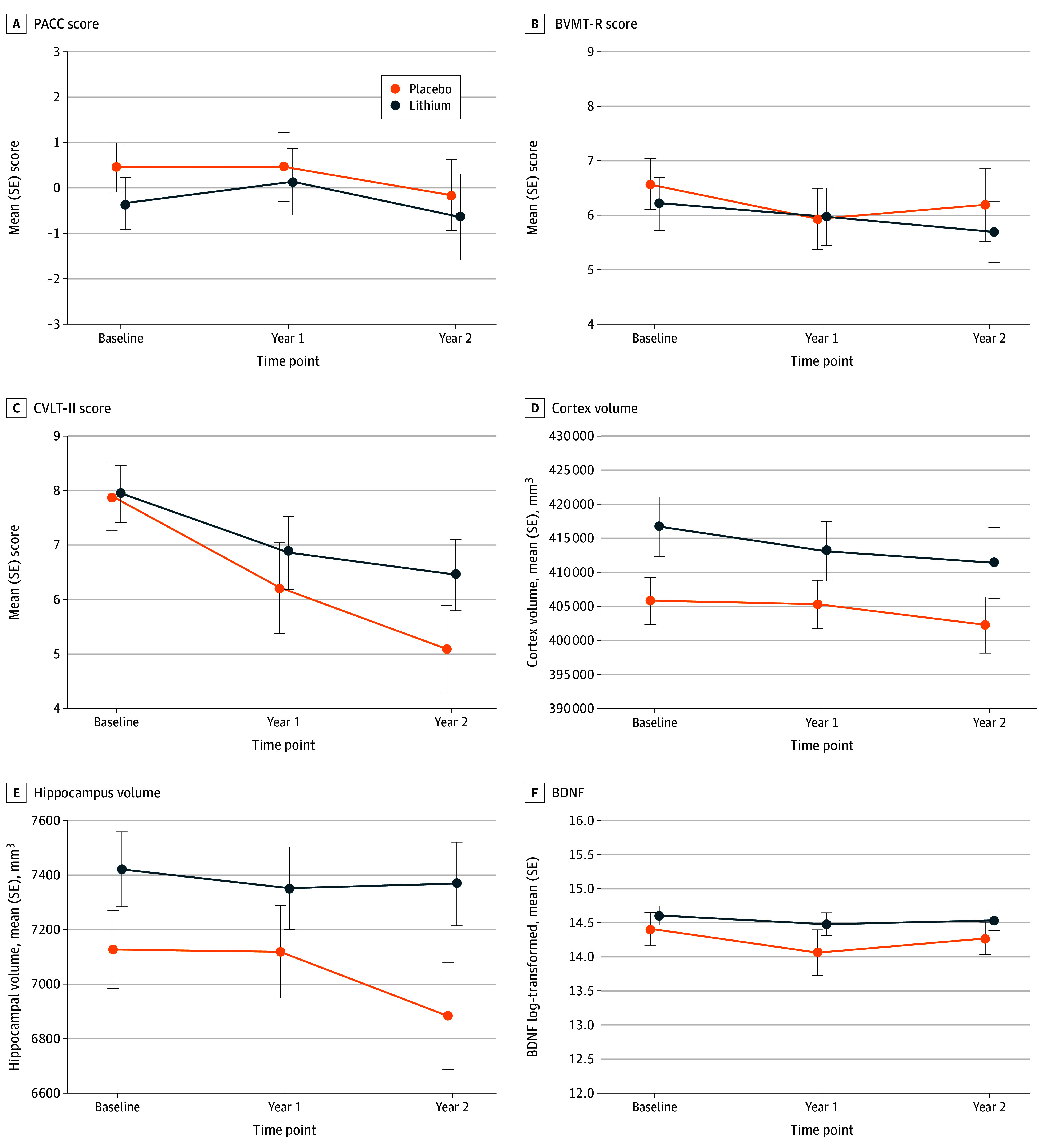
Line Graphs Showing Cognitive, Neuroimaging, and Brain-Derived Neurotrophic Factor (BDNF) Trajectories by Treatment Group Mean scores over 2 years for Preclinical Alzheimer Cognitive Composite (PACC) (A), Brief Visuospatial Memory Test-Revised (BVMT-R) delayed recall (B), California Verbal Learning Test-Second Edition (CVLT-II) delayed recall (C), cortical gray matter volume (cortex volume) (D), hippocampal volume (E), and BDNF (F) in lithium vs placebo groups. Error bars represent SEs.

We analyzed 6 coprimary outcomes using mixed-effects models: 3 cognitive (CVLT-II, BVMT-R, and PACC), 2 neuroimaging (cortex volume and hippocampal volume), and 1 biomarker (BDNF). Mean trajectories by treatment group are provided in Figure 3, and model results are summarized in Table 2. Significant declines during the study period were observed for CVLT-II and both neuroimaging measures. For the primary treatment comparisons (treatment × time interaction), only CVLT-II reached nominal significance (difference in annual decline, 0.69 points per year; 95% CI, 0.01-1.37; *P* = .05), which did not meet the prespecified threshold of *P* < .01.

**Table 2.** Mixed-Effects Model Results for Cognitive, Neuroimaging, and Biomarker Outcomes

Effect	Estimate (SE)	*df*	*t* Value	95% CI	*P* value
**PACC**					
Intercept	0.50 (0.61)	75	0.81	−0.73 to 1.72	.42
Intercept offset (lithium)^a^	−0.77 (0.84)	75	−0.92	−2.44 to 0.90	.36
Time (y)	−0.41 (0.26)	68	−1.60	−0.92 to 0.10	.11
Time × treatment^b^	0.087 (0.34)	68	0.25	−0.59 to 0.77	.80
**BVMT-R**					
Intercept	6.49 (0.47)	77	13.74	5.55 to 7.43	<.001
Intercept offset (lithium)^a^	−0.28 (0.66)	77	−0.42	−1.60 to 1.04	.68
Time (y)	−0.19 (0.23)	70	−0.83	−0.65 to 0.27	.41
Time × treatment^b^	−0.088 (0.31)	70	−0.29	−0.71 to 0.53	.78
**CVLT-II**					
Intercept	7.92 (0.61)	77	12.88	6.69 to 9.14	<.001
Intercept offset (lithium)^a^	−0.051 (0.86)	77	−0.06	−1.77 to 1.67	.95
Time (y)	−1.42 (0.25)	70	−5.67	−1.92 to −0.92	<.001
Time × treatment^b^	0.69 (0.34)	70	2.01	0.005 to 1.37	.05^c^
**Cortex volume, mm^3^**					
Intercept	407 162 (4045)	61	100.67	399 074 to 415 299	<.001
Intercept offset (lithium)^a^	9699 (5582)	61	1.74	−1463 to 20 862	.09
Time (y)	−2420 (939)	54	−2.58	−4303 to −536	.01
Time × treatment^b^	−352 (1254)	54	−0.28	−2866 to 2162	.78
**Hippocampus volume, mm^3^**					
Intercept	7166 (148)	61	48.51	6870 to 7462	<.001
Intercept offset (lithium)^a^	259 (204)	61	1.27	−148 to 667	.21
Time (y)	−121 (25)	54	−4.78	−172 to −70	<.001
Time × treatment^b^	59 (34)	54	1.74	−9 to 127	.09
**BDNF, ng/mL**					
Intercept	14.41 (0.19)	78	75.70	14.03 to 14.79	<.001
Intercept offset (lithium)^a^	0.18 (0.26)	78	0.70	−0.34 to 0.71	.49
Time (y)	−0.058 (0.10)	68	−0.57	−0.26 to 0.14	.57
Time × treatment^b^	0.012 (0.14)	68	0.09	−0.27 to 0.29	.93

Abbreviations: BDNF, brain-derived neurotrophic factor; BVMT-R, Brief Visuospatial Memory Test-Revised; CVLT-II, California Verbal Learning Test-II; df, degrees of freedom; PACC, Preclinical Alzheimer Cognitive Composite.

^a^ Intercept offsets represent differences in fitted lithium baseline means from placebo means.

^b^ Time × treatment effects represent the primary treatment comparisons. 95% CIs calculated using t distribution with appropriate *df*.

^c^ Nominally significant at α = .05 but does not meet the prespecified significance threshold (*P* < .01) used for the 6 coprimary outcomes.

Hippocampal volume decline was not significantly different between groups (difference in annual decline, 59 mm^3^ per year; 95% CI, −9 to 127; *P* = .09) (Table 2). Cortical gray matter volume showed no treatment × time interaction (difference in annual decline, −352 mm^3^ per year; 95% CI, −2866 to 2162; *P* = .78). BVMT-R, PACC, and BDNF showed no significant treatment × time interactions.

We performed prespecified analyses of participants who completed the study (eTable 4-6 in Supplement 2). Results in the overall completer sample (including both Aβ+ and Aβ-negative [Aβ−] participants) were consistent with the intention-to-treat analysis. In exploratory subgroup analyses stratified by amyloid status, sample sizes were limited. Effect sizes in Aβ+ completers were Hedges *g* = 0.74 for verbal memory, *g* = 0.82 for hippocampal volume, and *g* = 0.81 for hippocampal percentage change, compared with *g* = 0.32, *g* = 0.09, and *g* = 0.12, respectively, in Aβ− completers.

## Discussion

This pilot randomized clinical trial established the feasibility of recruitment and retention and confirmed the safety and tolerability of low-dose lithium in older adults with MCI. It generated preliminary effect size estimates across cognitive, neuroimaging, and plasma biomarker measures to inform future adequately powered trials.

Among cognitive outcomes, CVLT-II showed the largest effect size. Scores declined 1.42 points per year in the placebo group compared with 0.73 points per year in the lithium group (difference in annual decline, 0.69 points per year; 95% CI, 0.01-1.37; *P* = .05), which did not meet our prespecified threshold (*P* < .01). Neither BVMT-R nor PACC showed significant change over time in either group, limiting interpretation of treatment effects on these measures. The absence of decline may reflect insufficient sensitivity of these measures to detect change in this MCI population during a 2-year period.

For neuroimaging outcomes, both cortical gray matter and hippocampal volumes declined over time in both treatment groups. For hippocampal volume, the difference in decline between the groups did not reach statistical significance. BVMT-R, PACC, cortical gray matter volume, and BDNF showed no significant treatment × time interactions.

Despite 36% medication discontinuation overall (34% lithium vs 38% placebo), retention for outcome assessments exceeded 80%, meeting our feasibility target. Also, there was no statistical difference in the pattern of early discontinuation between the 2 groups, although power to detect differences was limited. Regarding safety, there were no serious adverse events definitely related to study medication, and the 1 death in the study occurred in the placebo group.

Conducting this trial provided knowledge and experience to inform future trials of low-dose lithium in MCI. Most importantly, we observed that older adults have substantial difficulty tolerating doses greater than 300 mg daily. Thus, future trials should use doses of lithium carbonate in the range of 150 mg to 300 mg daily.

Among prior RCTs of lithium in older adults with MCI and AD, results have varied based on treatment duration.^3,5,7,10^ Shorter-term trials^5,10^ (10-12 weeks) generally found no cognitive benefit, while longer-term trials^3,7^ (15-24 months), including ours, found effects on specific cognitive measures. These findings suggest that to detect cognitive effects in MCI, clinical trials need to be much longer than 10 to 12 weeks to observe a change in a declining trajectory.

### Limitations

Our study had some limitations. The RCT occurred during the COVID-19 pandemic, which affected in-person assessments and may have influenced medication discontinuation. Additionally, when the study was designed and launched between 2016 and 2017, we enrolled participants with syndromic MCI without requiring biomarker confirmation of AD pathology. At that time, screening for biological evidence of Alzheimer-type neurodegeneration was limited to amyloid PET imaging and lumbar puncture for cerebrospinal fluid analysis. Practical constraints for a pilot study led to this design decision: amyloid PET imaging was prohibitively expensive, and plasma biomarkers were unavailable. Our sample predominantly included participants with amnestic MCI but were Aβ−. This reflects our recruitment from the general community rather than memory disorders clinics, where prodromal AD is more prevalent.^49^ It may have diluted treatment effects. Exploratory analyses suggested larger effect sizes in Aβ+ participants, though small subgroup sizes limit interpretation. Trials can now use plasma-based biomarkers (eg, p-tau217) to enroll participants with AD pathology, thereby increasing statistical power.

## Conclusion

This pilot randomized clinical trial established the feasibility of recruitment and retention and confirmed the safety and tolerability of low-dose lithium treatment in older adults with MCI. The trial generated preliminary effect size estimates across cognitive and neuroimaging outcomes. Together with findings from prior independent longer-term trials, these results support further investigation of lithium in adequately powered trials to assess its potential neuroprotective properties in MCI.

## Notes

Supplement 1.
Trial Protocol

Supplement 2. **eTable 1**. Study Assessment Schedule and Instruments **eTable 2**. Serious Adverse Events by Treatment Group **eTable 3**. Summary of Adverse Events by Organ System and Treatment Group **eTable 4**. Individual Participant Changes from Baseline to 2 Years (Completers Analysis) **eTable 5**. Individual Participant Changes From Baseline to 2 Years in Aβ+ Participants (Completers Analysis) **eTable 6**. Individual Participant Changes from Baseline to 2 Years in Aβ− Participants (Completers Analysis)

Supplement 3.
Data Sharing Statement
