# Supplementary material for: Low-Dose Lithium for Mild Cognitive Impairment: A Pilot Randomized Clinical Trial
Source: JAMA Neurol. 2026 Mar 2;83(4):310–9. doi: 10.1001/jamaneurol.2026.0072 (PMC12954601; doi:10.1001/jamaneurol.2026.0072)
Supplement: Supplement 1. — Trial Protocol [file jamaneurol-e260072-s001.pdf]

Study Protocol

Official Title: Lithium As a Treatment to Prevent Impairment of Cognition in Elders (LATTICE)

ClinicalTrials.gov ID (NCT number): NCT03185208

Protocol Date: March 12, 2025

TABLE OF CONTENTS

Administrative Information
1. Descriptive title
2a. Trial identifier and registry name
3. Protocol version
4. Sources and types of financial, material, and other support
5a. Names, affiliations, and roles of protocol contributors
5b. Name and contact information for the trial sponsor
5c. Role of study sponsor and funders
Introduction
6a. Background
6b. Rationale
7. Objectives
8. Trial design
Methods: Participants, Interventions, Outcomes
9. Eligibility criteria
10. Study setting
11a. Description of interventions
11b. Explanation for choice of intervention
11c. Strategies to improve/monitor adherence
11d. Relevant concomitant care and interventions
12. Outcomes
13. Participant timeline
14. Sample size
15. Recruitment
Methods: Assignment of Interventions
16a. Allocation: Sequence generation
16b. Allocation: Concealment mechanism
16c. Allocation: Implementation
16d. Blinding
Methods: Data Collection, Management, and Analysis
17a. Data collection timing
17b. Data collection methods
18. Statistical methods
19. Methods for handling missing data
20a. Analysis population
20b. Methods for additional analyses
20c. Definition of analysis population
Methods: Monitoring
21a. Data monitoring committee
22. Harms
Ethics and Dissemination
24. Research ethics approval
25. Protocol amendments
26a. Consent/assent

26b. Additional consent provisions for biological specimens/ancillary studies
27. Confidentiality
29. Access to data
30. Ancillary and post-trial care
31a. Dissemination policy
33. Biological specimens

References cited

Appendix A: Statistical Analysis Plan

Administrative Information

1. Descriptive title: Lithium as a Treatment to Prevent Impairment of Cognition in Elders (LATTICE)

2a. Trial identifier and registry name: ClinicalTrials.gov identifier: NCT03185208.

3. Protocol version: The LATTICE Manual of Procedures is Version 1.6 2021-12-16.

4. Sources and types of financial, material, and other support: The study is supported by NIA Grant # R01AG055389-01.

5a. Names, affiliations, and roles of protocol contributors: The Principal Investigator is Ariel Gildengers, MD, affiliated with the Department of Psychiatry at the University of Pittsburgh.

5b. Name and contact information for the trial sponsor: The Institution/Sponsor is the University of Pittsburgh School of Medicine. Contact information for the PI, Dr. Gildengers, is provided in ClinicalTrials.gov.

5c. Role of study sponsor and funders: The NIA is the funder. The DSMB, which reports to the NIA, reviews the protocol, progress, participant risk/benefit, and can recommend continuation, termination, or modifications. The University of Pittsburgh IRB must approve the protocol and changes. The specific role of the funder/sponsor in data analysis, interpretation, report writing, or publication decisions is not explicitly detailed in these excerpts.

Introduction

6a. Background: Alzheimer's disease (AD) is the most common cause of dementia and its anticipated epidemic proportions. There is a lack of interventions with clear effects on AD progression and increasing interest in repurposing lithium for neurodegeneration. Lithium's potential mechanisms, such as promoting neurogenesis, up-regulating neurotrophic factors (Bcl-2, BDNF), and inhibiting GSK-3 (which plays roles in Aβ production and tau phosphorylation), are detailed below. Observational reports suggest lithium may delay dementia onset, but human trial results have been mixed.

There is evidence that lithium can have beneficial effects on cognition and brain health *in patients with bipolar disorder*.(1, 2) For example, long-term lithium treatment is associated with increased total and regional (fronto-limbic) gray matter.(3) Further, compared with patients not treated with lithium, studies have shown lithium treatment is associated with higher amygdala(4, 5) and hippocampal volumes(6, 7) and lower white matter microstructural abnormalities.(8) However, much more definitive findings are needed to recommend lithium's use more broadly in individuals in pre-symptomatic or mildly symptomatic AD because of its risk/benefit profile (i.e., narrow

therapeutic window with risk for neurological or renal toxicity). Should lithium prove to have anti-AD properties with a favorable risk/benefit profile, it can deployed rapidly and broadly because it is inexpensive and already commercially available.

While a comprehensive discussion of lithium's effects on the brain and cognition is beyond the scope of this protocol, we summarize key findings supporting this study and refer to reviews addressing this topic.(3, 9-13) Briefly, various lines of evidence point to the beneficial effects of lithium, including molecular and cellular data,(14) rodent and human data,(15-18) and epidemiologic data.(19, 20) **Table 1** summarizes lithium's known *in vitro* and *in vivo* neuro-cognitive effects in rodents and humans.(9) On a cellular level, lithium inhibits GSK-3 (isoforms α and β), an enzyme that directly modulates cell survival, synaptic plasticity, cellular structure and resilience.(21) In particular, GSK-3α is involved in maximal processing of APP to Aβ; GSK-3β phosphorylates tau.(22) Additionally, through downstream effects of GSK-3 inhibition, lithium is related to up-regulation of various neurotrophic factors, including BDNF and Bcl-2 that may enhance brain health and long-term cognitive function.(10, 21, 23-25) In particular, lithium treatment may be related hippocampal neurogenesis.(3, 26) **Figure 1** below, taken from a review on the neuroprotective effects of lithium for ischemic stroke illustrates these cellular effects.(27) <u>*Hence, through multiple pathways, it appears that long-term lithium use may help preserve brain health and cognitive function*</u>.

Table 1. Neurotrophic and neuroprotective effects of lithium[9]

**Protects (human and rodent) brain cells in vitro from**
Glutamate and NMDA toxicity
Calcium toxicity
Thapsigargin (which mobilizes MPP^+ and Ca^{2+}) toxicity
β-amyloid toxicity
Aging-induced cell death
Growth factor and serum deprivation
Glucose deprivation
Low K^+
C2-ceramide
Ouabain
Aluminum toxicity
HIV regulatory protein, Tat
**Effects in rodent brain (in vivo)**
Enhanced hippocampal neurogenesis
Protection against cholinergic lesions
Protection against radiation injury
Protection against medial cerebral artery occlusion (stroke model)
Protection against quinolinic acid (Huntington's model)
**Effects in human brain**
Increased gray matter volumes in lithium-treated bipolar patients
Increased N-acetylaspartate (NAA) levels in lithium-treated BD pts
Protection against reduced subgenual prefrontal cortex volumes
Larger anterior cingulate volumes in lithium-treated bipolar patients
Protection against reduced glial #s or glia:neuron ratio in the amygdala

6b. Rationale: Research is needed to determine if lithium has a role as an anti-dementia agent. This pilot-feasibility study aims to examine lithium's potential disease modifying properties in individuals with mild cognitive impairment (MCI). The study uses a more integrative, comprehensive approach than previous trials, incorporating state-of-the-art techniques like 7T MRI, neurocognitive assessment, and blood/CSF biomarkers.

Figure 1. Cellular effects of lithium[27]

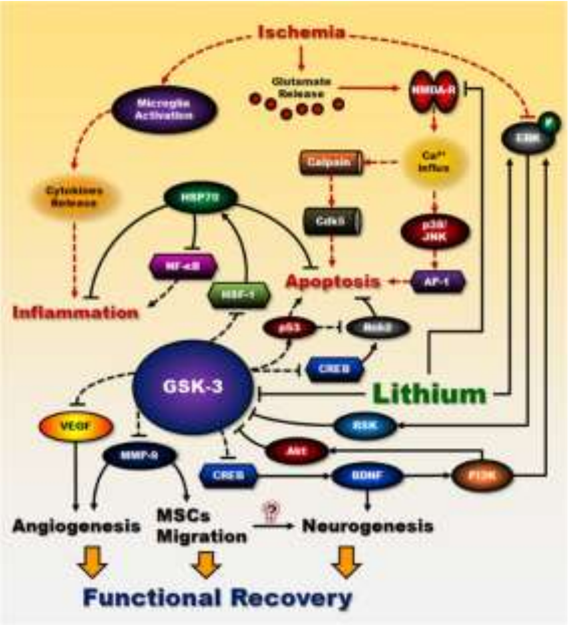

7. Objectives: The Specific Aim is to examine the potential disease modifying properties of lithium in individuals with MCI in delaying conversion to dementia. The Exploratory Aim is to examine whether lithium is related to additional markers of enhanced brain integrity (e.g., lower microbleeds, higher white matter integrity, better connectivity, decreased CSF phospho tau).

8. Trial design: This is a two-year, double-blind, randomized controlled trial comparing lithium to placebo in individuals with MCI. It is a pilot-feasibility study. Participants are randomly assigned based on a permuted block design stratified by baseline PiB PET status (amyloid positive/negative/undetermined). The study involves comprehensive assessments including ultra-high field (7T) human MRI, neurocognitive assessment, blood- and CSF-based biomarker measurement, and Aβ imaging at baseline, with follow-up assessments annually.

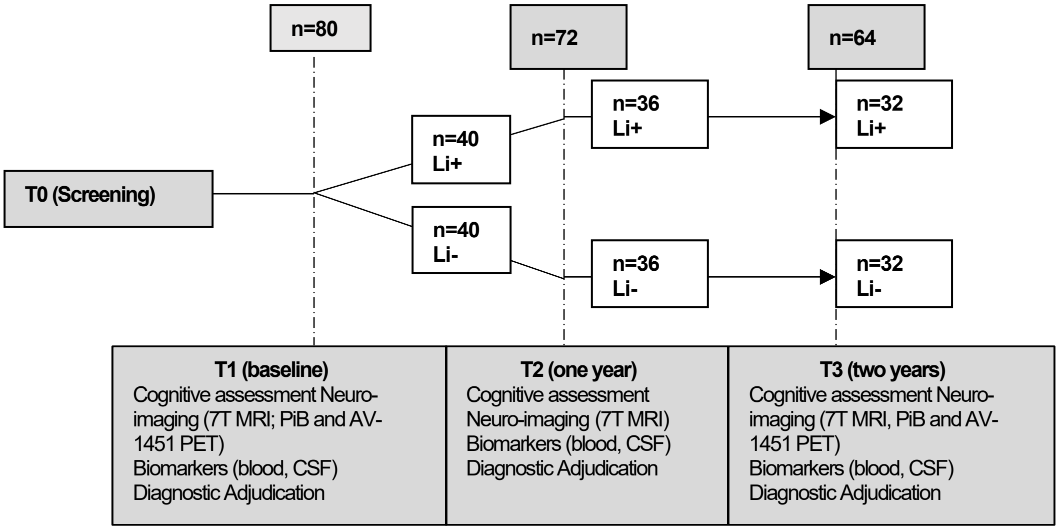

Methods: Participants, Interventions, Outcomes

9. Eligibility criteria:

Inclusion Criteria: Ages 60 and above; Diagnosis of Mild Cognitive Impairment.

Exclusion Criteria: Major psychiatric illness (mild illness without current pharmacotherapy may be included); Major neurologic illness; Contraindication to lithium (e.g., renal insufficiency); Unable to complete neuropsychological testing due to non-remediable impairment; Any clinically unstable systemic illness.

10. Study setting: The study is conducted at the University of Pittsburgh School of Medicine. Locations include Western Psychiatric Institute and Clinic, Oxford Building, UPMC Presbyterian (PET Facility, Magnetic Resonance Research Center - MRRC), UPMC Montefiore (Investigational Drug Service - IDS), Benedum Geriatric Center, and Quest facilities for labs.

11a. Description of interventions: Participants are randomly assigned to receive either lithium or placebo. The study drug (lithium or placebo) is compounded and over-encapsulated into identical capsules by the IDS for blinding. Participants start at a low dose (150 mg) and the dose is titrated up to a target blood level of 0.6 to 0.8 mEq/L. Titration involves checking lithium levels and adjusting the dose. Pills come in 150 mg, 300 mg, or 450 mg doses. At the end of the study (Year 2), participants are tapered off the study drug over four weeks by decreasing the dose by 150 mg per week.

11b. Explanation for choice of intervention: The rationale is based on lithium's potential neurobiological effects relevant to AD pathology (Aβ and tau), evidence from animal models and some human studies (observational and in bipolar disorder), suggesting it might alter the AD trajectory.

11c. Strategies to improve/monitor adherence: Participants receive study drug dispensed for periods (initially 2 weeks, then quarterly supply). An emergency supply is provided. Dispensing and accountability logs are kept by IDS and research staff. Lithium levels are checked bi-weekly during titration, then quarterly and annually, to monitor compliance and safety. Phone calls are made monthly to administer the BARS (Brief Adherence Rating Scale) to assess adherence. Weekly check-in calls during titration ask about side effects. Study partners may be enlisted to help with adherence.

11d. Relevant concomitant care and interventions: Participants may continue all medications prescribed for their routine medical care. Mild psychiatric illness is not an exclusion if there is no current pharmacotherapy. Special consideration is given to patients on NSAIDs and Thiazide Diuretics during lithium dose adjustment due to potential interactions.

12. Outcomes:

Primary Outcomes: Cognitive function (primarily memory) and neuroimaging measures (larger hippocampal volumes and lower total gray matter thinning). PACC scores, CVLT-II and BVMT-R are main cognitive outcome measures.

Secondary Outcomes: Changes in specific biomarkers (e.g., GSK-3β activity, BDNF) associated with changes in cognition and neuroimaging.

Exploratory Outcomes: Changes in additional markers of brain integrity (e.g., microbleeds, white matter integrity (FA), network connectivity, CSF phospho tau), other CSF biomarkers (Aβ, total tau), blood biomarkers.

Time points: Outcomes are measured at Baseline (T1: T1a, T1b, T1c), 1 Year (T2), and 2 Years (T3). Safety labs and lithium levels are obtained quarterly and annually. Cognitive assessments, neuroimaging (MRI), and blood/CSF biomarkers are done at baseline, Year 1, and Year 2. PiB scans are done at baseline and Year 2.

13. Participant timeline: A detailed schedule outlines visits from screening (T0), baseline (T1a-c), weekly titration visits/calls, monthly phone visits, quarterly in-person visits (Q1a-c, Q2a-c), annual visits (T2, T3), and tapering. The total duration of lithium/placebo treatment is two years. Recruitment and subject completion timelines span across Y01-Y04.

14. Sample size: The study targets enrolling 80 subjects, anticipating a 20% attrition rate to achieve approximately 64 completers. This sample size is powered to detect a medium effect size (Cohen's d of 0.57) for primary outcomes over three time points with repeated measures modeling.

15. Recruitment: Recruitment methods include presentations on "cognitive aging" at local facilities for older adults, offering brief cognitive screening and study information. This network of facilities and advertising materials yields audiences. Local talks are supplemented with radio, bus, and senior newspaper advertisements, and recruitment from university research registries.

Methods: Assignment of Interventions (for controlled trials)

16a. Allocation: Sequence generation: Randomization lists are generated by the statistician using a permuted block design.

16b. Allocation: Concealment mechanism: The master randomization lists are stored in a database accessible only to unblinded individuals.

16c. Allocation: Implementation: Research staff (who are blinded) notify the Data Manager (Happy Fletcher), who is unblinded, that a participant is eligible. The research staff provide the participant's ID number and PiB category. The Data Manager then randomizes the subject and informs the Investigational Drug Service (IDS) and the Blinded Labs Manager.

16d. Blinding: The study is double-blind; neither the participants nor the research staff/physicians prescribing the pills know if they contain lithium or placebo. The IDS prepares identical capsules for lithium and placebo to maintain blinding. A Blinded Labs Manager reviews true lithium levels and provides a false level to the blinded research team for participants on placebo, calculated to mimic possible lithium levels. Unblinded investigators/staff are available for emergencies. The blind is broken to the treating doctor (not the research team) at the end of the study if they plan to continue prescribing.

Methods: Data Collection, Management, and Analysis

17a. Data collection timing: Data is collected at specific visits: T0 (Screening), T1a-c (Baseline), weekly titration, monthly (M2, M4, M5, M7, M8, M10, M11, M13, M14, M16, M17, M19, M20, M22, M23), quarterly (Q1a-c, Q2a-c), annual (T2, T3), and termination/tapering. Specific assessments (cognitive, imaging, labs, biomarkers) occur at defined time points as detailed in the schedule.

17b. Data collection methods: Data is initially collected on paper forms (CRFs). These forms are then entered into an electronic database on secure, password protected servers. Paper copies are retained in locked file cabinets. Cognitive

assessments are administered by trained research staff. Blood draws are done by trained phlebotomists. Lumbar punctures for CSF are done by a trained neurologist or physician. Neuroimaging is performed by trained and experienced staff. Quality control procedures include written procedures, checklists, and training for staff. Data entry corrections are tracked. Data Feedback Forms document assessment status and missing data codes.

18. Statistical methods: Analyses will start with basic descriptive techniques and two-sample tests (t-tests, Wilcoxon tests, chi-square tests). Linear mixed effects models will be used for the primary analysis of outcomes measured at three time points (time, group, and time-by-group interaction terms). Potential and exploratory covariates will be included. Tobit models may be used for biomarker results outside detectable limits. Subgroup analyses will use mixed models with subgroup and interaction terms. Multi-block Partial Least Squares (PLS) will integrate structural MR, fMRI, PET, and cognitive data. Statistical significance will be determined using permutation testing, and reliability using bootstrapping ratios. Analyses are two-sided, interpreted at the 0.05 level, with adjusted analysis using $p<0.01$ and FWE correction for voxel-wise analyses.

19. Methods for handling missing data: The analyses will assess the impact of missing data. If missingness is random, inverse probability weighting or imputation will be used. Missing codes (e.g., Not applicable, Assessment done on phone) are utilized to categorize reasons for missing data. The Data Manager generates reports on data completeness.

20a. Analysis population: The study will follow intention to treat principles, analyzing subjects according to their original randomized assignment, even if they do not complete the intervention.

20b. Methods for additional analyses: Subgroup analyses (e.g., APOE-ε4, aMCI multiple domains, PiB+) using mixed models are planned. Integration of neuroimaging and cognitive data using multi-block PLS is planned. Adjusted analyses including covariates are described.

20c. Definition of analysis population: See 20a. Statistical methods for handling missing data are described (inverse probability weighting, imputation).

Methods: Monitoring

21a. Data monitoring committee: A Data Safety Monitoring Board (DSMB) is impaneled with external experts in relevant fields. The DSMB convenes twice a year and as needed. Its role includes evaluating trial progress, data quality/timeliness, recruitment/retention, participant risk versus benefit, site performance, and

considering external factors. The DSMB recommends continuation, termination, or modifications to the trial.

22. Harms: Adverse events (AEs), serious adverse events (SAEs), unanticipated problems, and protocol deviations are monitored. SAEs are reported immediately to the PI, DSMB/NIA, and Pitt IRB. AE/Unanticipated Problems are reviewed at weekly data and safety monitoring meetings and reported quarterly to the NIA Program Office and DSMB. Lithium levels are monitored frequently during titration and quarterly/annually for safety. Baseline and follow-up safety labs (basic metabolic panel, TSH) are reviewed by a physician-investigator, and clinically significant changes are reported as AEs. Discovery of unexpected or frequent AEs triggers reporting and potential modification or termination of the study.

Ethics and Dissemination

24. Research ethics approval: Prospective approval of the protocol, informed consent forms, modifications, and advertisements is obtained from the University of Pittsburgh Institutional Review Board (IRB). The IRB operates in compliance with FDA regulations and ICH GCP guidelines.

25. Protocol amendments: Important protocol modifications require prospective IRB approval. A Decision Log is used to track modifications to the MOP and decisions made by investigators/regulatory bodies. Previous versions of the MOP are retained. Participants are re-consented to modified protocols before additional procedures are performed.

26a. Consent/assent: Verbal informed consent is obtained for screening procedures after being read a script, as a waiver has been granted by the IRB. Written informed consent is obtained by a physician-investigator prior to the baseline visit (T1a) using the main informed consent document. Consent is viewed as an ongoing process. An X-ray addendum consent is reviewed if needed.

26b. Additional consent provisions for biological specimens/ancillary studies: Informed consent documents include language allowing the sharing of de-identified participant data and coded biological samples (blood, CSF) with the NIH and other qualified investigators (via the National Cell Repository for Alzheimer Disease - NCRAD) for research purposes, including potential genetic analysis and studies of other diseases. Samples are stored indefinitely for long-term research. Successful research could lead to commercial products, but participants will not share financial benefits.

27. Confidentiality: Personal identifying information (name, DOB) is kept separate from research data (linked by ID number) and destroyed if the patient does not consent to the study. Data is entered into an electronic database on secure,

password protected servers with limited access. Paper copies are kept in locked file cabinets. The Blinded Labs Manager handles lithium levels using ID numbers to protect blinding for the research team, and true levels do not appear in the patient's clinical chart linked to their name/MRN. De-identified data may be shared. DSMB ensures confidentiality of study data.

29. Access to data: Access to the electronic database is limited to research staff and investigators. Access to master randomization lists is limited to unblinded individuals. A final dataset is provided to investigators after database freeze. Data (and samples) are made available to the NIA and other qualified investigators through NCRAD after identifying linkage is destroyed.

30. Ancillary and post-trial care: Participants whose cognitive scores indicate AD may be referred to the Alzheimer Disease Research Center for further evaluation and treatment. Participants with a clinical caretaker who wishes to continue lithium treatment after the trial may be provided an appropriate supply. No information is provided on compensation for potential harm from trial participation. Participants are not responsible for study costs.

31a. Dissemination policy: The study will be registered with ClinicalTrials.gov. Findings will be reported following CONSORT Guidelines. There is a plan to share data and biological materials with the NIH and qualified investigators through NCRAD.

33. Biological specimens: Samples are processed, labeled with ID numbers, and frozen for later batch analysis. Samples are stored in the Geriatric Neuropsychopharmacology Lab. Plans for future use in ancillary studies and sharing with NCRAD are included in the consent and procedures. Procedures for shipping samples to NCRAD are detailed.

References cited

1. Rybakowski JK. Lithium in neuropsychiatry: a 2010 update. World J Biol Psychiatry. 2011;12(5):340–8.
2. Lan CC, Liu CC, Lin CH, Lan TY, McInnis MG, Chan CH, et al. A reduced risk of stroke with lithium exposure in bipolar disorder: a population-based retrospective cohort study. Bipolar Disord. 2015;17(7):705–14.
3. Malhi GS, Tanious M, Das P, Coulston CM, Berk M. Potential mechanisms of action of lithium in bipolar disorder. Current understanding. CNS Drugs. 2013;27(2):135–53.
4. Usher J, Menzel P, Schneider-Axmann T, Kemmer C, Reith W, Falkai P, et al. Increased right amygdala volume in lithium-treated patients with bipolar I disorder. Acta Psychiatr Scand. 2010;121(2):119–24.
5. Foland LC, Altshuler LL, Sugar CA, Lee AD, Leow AD, Townsend J, et al. Increased volume of the amygdala and hippocampus in bipolar patients treated with lithium. Neuroreport. 2008;19(2):221–4.
6. Hajek T, Cullis J, Novak T, Kopecek M, Hoschl C, Blagdon R, et al. Hippocampal volumes in bipolar disorders: opposing effects of illness burden and lithium treatment. Bipolar Disord. 2012;14(3):261–70.
7. Hajek T, Kopecek M, Hoschl C, Alda M. Smaller hippocampal volumes in patients with bipolar disorder are masked by exposure to lithium: a meta-analysis. J Psychiatry Neurosci. 2012;37(3):110143.
8. Macritchie KA, Lloyd AJ, Bastin ME, Vasudev K, Gallagher P, Eyre R, et al. White matter microstructural abnormalities in euthymic bipolar disorder. Br J Psychiatry. 2010;196(1):52–8.
9. Schloesser RJ, Huang J, Klein PS, Manji HK. Cellular plasticity cascades in the pathophysiology and treatment of bipolar disorder. Neuropsychopharmacology. 2008;33(1):110–33.
10. Schloesser RJ, Martinowich K, Manji HK. Mood-stabilizing drugs: mechanisms of action. Trends Neurosci. 2012;35(1):36–46.
11. Matsunaga S, Kishi T, Annas P, Basun H, Hampel H, Iwata N. Lithium as a Treatment for Alzheimer's Disease: A Systematic Review and Meta-Analysis. J Alzheimers Dis. 2015;48(2):403–10.
12. Forlenza OV, Aprahamian I, de Paula VJ, Hajek T. Lithium, a therapy for AD: current evidence from clinical trials of neurodegenerative disorders. Curr Alzheimer Res. 2016.
13. Forlenza OV, de Paula VJ, Machado-Vieira R, Diniz BS, Gattaz WF. Does lithium prevent Alzheimer's disease? Drugs Aging. 2012;29(5):335–42.
14. Quiroz JA, Gould TD, Manji HK. Molecular effects of lithium. Mol Interv. 2004;4(5):259–72.
15. Moore GJ, Bebchuk JM, Hasanat K, Chen G, Seraji-Bozorgzad N, Wilds IB, et al. Lithium increases N-acetyl-aspartate in the human brain: in vivo evidence in support of bcl-2's neurotrophic effects? Biol Psychiatry. 2000;48(1):1–8.
16. Moore GJ, Bebchuk JM, Wilds IB, Chen G, Manji HK. Lithium-induced increase in human brain grey matter. Lancet. 2000;356(9237):1241–2.
17. Bachmann RF, Wang Y, Yuan P, Zhou R, Li X, Alesci S, et al. Common effects of lithium and valproate on mitochondrial functions: protection against

methamphetamine-induced mitochondrial damage. Int J Neuropsychopharmacol. 2009;12(6):805–22.
18. Chen G, Zeng WZ, Yuan PX, Huang LD, Jiang YM, Zhao ZH, et al. The mood-stabilizing agents lithium and valproate robustly increase the levels of the neuroprotective protein bcl-2 in the CNS. J Neurochem. 1999;72(2):879–82.
19. Kessing LV, Sondergard L, Forman JL, Andersen PK. Lithium Treatment and Risk of Dementia. Arch Gen Psychiatry. 2008;65(11):1331–5.
20. Gerhard T, Devanand DP, Huang C, Crystal S, Olfson M. Lithium treatment and risk for dementia in adults with bipolar disorder: population-based cohort study. Br J Psychiatry. 2015.
21. Machado-Vieira R, Soeiro-De-Souza MG, Richards EM, Teixeira AL, Zarate CA, Jr. Multiple levels of impaired neural plasticity and cellular resilience in bipolar disorder: developing treatments using an integrated translational approach. World J Biol Psychiatry. 2014;15(2):84–95.
22. Phiel CJ, Wilson CA, Lee VM, Klein PS. GSK-3alpha regulates production of Alzheimer's disease amyloid-beta peptides. Nature. 2003;423(6938):435–9.
23. Berk M, Conus P, Kapczinski F, Andreazza AC, Yucel M, Wood SJ, et al. From neuroprogression to neuroprotection: implications for clinical care. Med J Aust. 2010;193(4 Suppl):S36–40.
24. Young W. Review of lithium effects on brain and blood. Cell Transplant. 2009;18(9):951–75.
25. Khairova R, Pawar R, Salvadore G, Juruena MF, de Sousa RT, Soeiro-de-Souza MG, et al. Effects of lithium on oxidative stress parameters in healthy subjects. Molecular medicine reports. 2012;5(3):680–2.
26. Gray JD, McEwen BS. Lithium's role in neural plasticity and its implications for mood disorders. Acta Psychiatr Scand. 2013;128(5):347–61.
27. Chuang DM, Wang Z, Chiu CT. GSK-3 as a Target for Lithium-Induced Neuroprotection Against Excitotoxicity in Neuronal Cultures and Animal Models of Ischemic Stroke. Front Mol Neurosci. 2011;4:15.

[redacted]

[redacted]

[redacted]

[redacted]

[redacted]

[redacted]

<u>DATA MANAGEMENT AND ANALYSIS</u>

<u>Oversight</u>. Stewart J. Anderson, Ph.D., Professor of Biostatistics, and Dana Tudorascu, Ph.D., Assistant Professor of Medicine and Biostatistics, will conduct or oversee all statistical analyses.

<u>Data Collection</u>. We will contract with the Center for Research on Health Care of the University of Pittsburgh to create an on-line, secure data management application. The on-line system will enable real-time data validation, data verification, and subject tracking.

<u>HYPOTHESIS TESTING</u>. All statistical analyses will be conducted starting with basic descriptive techniques and two sample testing (t-tests, Wilcoxon tests, chi-square tests) for testing the difference between the intervention and control groups. Statistical tests will be two-sided and interpreted at the 0.05 level. <u>We have also performed all of our power computations and have provided effect sizes for all adjusted analysis (p<0.01) due to multiple testing. Additionally, all of our voxel-wise analyses results will be corrected using FWE</u>. These analyses will then inform the next step in the analysis process that will include statistical modeling. First, adherence will be assessed over time by intervention group using a Kaplan-Meier approach where non-adherence is considered to be an "event." For all modeling, we will use regression diagnostics to identify potential outliers or influential observations. Additionally, the analyses will be assessed for the impact of missing data. For the initial reporting of all results, we will follow the guidelines outlined in the CONSORT statement and follow intention to treat principles: subjects will continue to be followed and analyzed according to the original randomized assignment.[134, 135] Tests of whether missingness is at random will be performed. Should missing data prove to be an issue, we will rely on inverse probability weighting or imputation as part of the analysis.

<u>Specific Aim: H1-2</u>. This analysis focuses on assessing the effect of lithium on: (H1) cognitive performance and, (H2) neuroimaging measures of hippocampal volumes and total gray matter and cognition. Outcomes are measured at three time points, and our primary analysis will consist of linear mixed effects models. These models will include a term for time, group and a time-by-group interaction. We will test for lithium effect by testing the significance of the coefficient associated with the interaction term in the model as well as by a likelihood ratio test between the models with and without interaction. First, we will fit these basic models for each of the outcomes of interest and test for the time-by-group interaction as an assessment of the effect of lithium. Second, we will include potential covariates (e.g., age, education, sex, etc.) and exploratory covariates (e.g., physical activity) that may influence the group effect and test for a time-by-group interaction in this context. We will examine the biomarker trajectories across the two groups. If the assays used to measure the biomarkers produce results

outside of the detectable limits, we will use tobit model and mixed model extensions of the tobit model to test for differences between the two groups.

Exploratory Aim and subgroup analyses. Following methods outlined above, we will examine whether lithium is related to additional changes in markers of brain integrity (e.g., cerebrovascular integrity, white matter integrity, functional connectivity; CSF Aβ, total tau, and phospho tau). Further, we will assess the effect of lithium in the following pre-specified subgroups: individuals who are APOE-ε4, aMCI (multiple domains), or PiB+. For each of these groups, we will fit mixed models with subgroup as a covariate and the subgroup and group interaction term. In addition, we will consider the application of newer methods for subgroup analysis as outlined in Tian, Alizadeh, Gentles and Tibshirani.[136] They discuss the use of a modified covariate for the testing of the interactions in one large model through the use of variable selection techniques. The results of this analysis may lead to potential subgroups for further study. In addition to individual ROI models, we will also integrate the structural MR, fMRI, and PET with cognitive function to explore differences between groups in the context of the interrelated structure of the neuroimaging modalities and cognitive function. These analyses will be performed using multi-block Partial Least Squares (**multi-block PLS**).[137] Multi-block PLS can analyze three or more matrices (MR, fMRI, PET, cognitive measures) simultaneously and identify the patterns of brain activity and measures related changes in cognitive functions, contrasts (time) across groups by using a singular value decomposition algorithm (**SVD**).[138, 139] Multi-table PLS will generate (via SVD) design latent variables that will show changes in brain activity between groups and brain related latent variables (saliences) that will characterize the relationships between imaging modalities. The statistical significance of these models will be obtained by using permutation testing and the reliability of the resulting latent variables will be determined using bootstrapping ratios (the ratio of the brain saliences to the bootstrap standard error). This ratio is approximately equivalent to a Z-score given a normal bootstrap distribution.[140]

POWER ANALYSIS. For H1a/H2a, where 80 subjects will be randomized to one of two groups with 64 completers (32 in each cell), we computed power based on the following assumptions: a two-sided significance level of 0.05, a power of 0.80 and measurements at three time points for a repeated measures model. Based on these assumptions, we are powered to observe an effect size (Cohen's **d**) of 0.57 (i.e., medium) when the observations are correlated at a level of 0.5 across time. The effect size ranges from 0.45 to 0.60, a little over a half standard deviation difference in the outcome variable, as the correlation between repeated observations ranges from 0.1 to 0.6. We also computed the effect size for a two-sided significance level of 0.01, *a more conservative alpha for testing multiple outcomes*, and power of 0.80 as above and found that the effect size ranged from 0.54 to 0.72 as the correlation between repeated observations ranged from 0.1 to 0.6.

[redacted]

[redacted]

[redacted]

BIBLIOGRAPHY AND REFERENCES CITED

1. Scheltens P, Blennow K, Breteler MM, de Strooper B, Frisoni GB, Salloway S, Van der Flier WM. Alzheimer's disease. Lancet. 2016. doi: 10.1016/S0140-6736(15)01124-1. PubMed PMID: 26921134.
2. NIH. PAR-16-365: Pilot Clinical Trials for the Spectrum of Alzheimer's Disease and Age-related Cognitive Decline (R01). 2015.
3. Chuang DM, Wang Z, Chiu CT. GSK-3 as a Target for Lithium-Induced Neuroprotection Against Excitotoxicity in Neuronal Cultures and Animal Models of Ischemic Stroke. Front Mol Neurosci. 2011;4:15. doi: 10.3389/fnmol.2011.00015. PubMed PMID: 21886605; PubMed Central PMCID: PMCPMC3152742.
4. Association As. Changing the Trajectory of Alzheimer's Disease 2015. Available from: http://www.alz.org/alzheimers_disease_trajectory.asp.
5. Lazzara CA, Kim YH. Potential application of lithium in Parkinson's and other neurodegenerative diseases. Front Neurosci. 2015;9:403. doi: 10.3389/fnins.2015.00403. PubMed PMID: 26578864; PubMed Central PMCID: PMCPMC4621308.
6. van Erp TG, Thompson PM, Kieseppa T, Bearden CE, Marino AC, Hoftman GD, Haukka J, Partonen T, Huttunen M, Kaprio J, Lonnqvist J, Poutanen VP, Toga AW, Cannon TD. Hippocampal morphology in lithium and non-lithium-treated bipolar I disorder patients, non-bipolar co-twins, and control twins. Human brain mapping. 2012;33(3):501-10. Epub 2011/04/02. doi: 10.1002/hbm.21239. PubMed PMID: 21455943.
7. Schloesser RJ, Martinowich K, Manji HK. Mood-stabilizing drugs: mechanisms of action. Trends Neurosci. 2012;35(1):36-46. Epub 2012/01/06. doi: S0166-2236(11)00196-2 [pii] 10.1016/j.tins.2011.11.009. PubMed PMID: 22217451.
8. Matsunaga S, Kishi T, Annas P, Basun H, Hampel H, Iwata N. Lithium as a Treatment for Alzheimer's Disease: A Systematic Review and Meta-Analysis. Journal of Alzheimer's disease : JAD. 2015;48(2):403-10. doi: 10.3233/JAD-150437. PubMed PMID: 26402004.
9. Phiel CJ, Wilson CA, Lee VM, Klein PS. GSK-3alpha regulates production of Alzheimer's disease amyloid-beta peptides. Nature. 2003;423(6938):435-9. Epub 2003/05/23. doi: 10.1038/nature01640 nature01640 [pii]. PubMed PMID: 12761548.
10. Gerhard T, Devanand DP, Huang C, Crystal S, Olfson M. Lithium treatment and risk for dementia in adults with bipolar disorder: population-based cohort study. The British journal of psychiatry : the journal of mental science. 2015. doi: 10.1192/bjp.bp.114.154047. PubMed PMID: 25614530.
11. Nunes PV, Forlenza OV, Gattaz WF. Lithium and risk for Alzheimer's disease in elderly patients with bipolar disorder. The British Journal of Psychiatry. 2007;190(4):359-60. doi: 10.1192/bjp.bp.106.029868.
12. Kessing LV, Forman JL, Andersen PK. Does lithium protect against dementia? Bipolar Disord. 2010;12(1):87-94. doi: 10.1111/j.1399-5618.2009.00788.x. PubMed PMID: 20148870.
13. Kessing LV, Sondergard L, Forman JL, Andersen PK. Lithium Treatment and Risk of Dementia. Archives of general psychiatry. 2008;65(11):1331-5. doi: 10.1001/archpsyc.65.11.1331.
14. Terao T, Nakano H, Inoue Y, Okamoto T, Nakamura J, Iwata N. Lithium and dementia: a preliminary study. Progress in neuro-psychopharmacology & biological psychiatry. 2006;30(6):1125-8. Epub 2006/06/07. doi: S0278-5846(06)00182-5 [pii] 10.1016/j.pnpbp.2006.04.020. PubMed PMID: 16753246.
15. Hampel H, Ewers M, Burger K, Annas P, Mortberg A, Bogstedt A, Frolich L, Schroder J, Schonknecht P, Riepe MW, Kraft I, Gasser T, Leyhe T, Moller HJ, Kurz A, Basun H. Lithium trial in Alzheimer's disease: a randomized, single-blind, placebo-controlled, multicenter 10-week study. The Journal of clinical psychiatry. 2009;70(6):922-31. PubMed PMID: 19573486.
16. Macdonald A, Briggs K, Poppe M, Higgins A, Velayudhan L, Lovestone S. A feasibility and tolerability study of lithium in Alzheimer's disease. International journal of geriatric psychiatry. 2008;23(7):704-11. doi: 10.1002/gps.1964. PubMed PMID: 18181229.
17. Forlenza OV, Diniz BS, Radanovic M, Santos FS, Talib LL, Gattaz WF. Disease-modifying properties of long-term lithium treatment for amnestic mild cognitive impairment: randomised controlled trial. The British journal of psychiatry : the journal of mental science. 2011;198(5):351-6. doi: 10.1192/bjp.bp.110.080044. PubMed PMID: 21525519.
18. Nunes MA, Viel TA, Buck HS. Microdose lithium treatment stabilized cognitive impairment in patients with Alzheimer's disease. Current Alzheimer research. 2013;10(1):104-7. PubMed PMID: 22746245.
19. Duara R, Loewenstein DA, Greig MT, Potter E, Barker W, Raj A, Schinka J, Borenstein A, Schoenberg M, Wu Y, Banko J, Potter H. Pre-MCI and MCI: neuropsychological, clinical, and imaging features and progression rates. The American journal of geriatric psychiatry : official journal of the American Association for Geriatric Psychiatry. 2011;19(11):951-60. doi: 10.1097/JGP.0b013e3182107c69. PubMed PMID: 21422909; PubMed Central PMCID: PMCPMC3175279.

20. Jack CR, Jr., Knopman DS, Jagust WJ, Shaw LM, Aisen PS, Weiner MW, Petersen RC, Trojanowski JQ. Hypothetical model of dynamic biomarkers of the Alzheimer's pathological cascade. Lancet neurology. 2010;9(1):119-28. doi: 10.1016/S1474-4422(09)70299-6. PubMed PMID: 20083042; PubMed Central PMCID: PMCPMC2819840.
21. Rybakowski JK. Lithium in neuropsychiatry: a 2010 update. The world journal of biological psychiatry : the official journal of the World Federation of Societies of Biological Psychiatry. 2011;12(5):340-8. doi: 10.3109/15622975.2011.559274. PubMed PMID: 21361856.
22. Lan CC, Liu CC, Lin CH, Lan TY, McInnis MG, Chan CH, Lan TH. A reduced risk of stroke with lithium exposure in bipolar disorder: a population-based retrospective cohort study. Bipolar Disord. 2015;17(7):705-14. doi: 10.1111/bdi.12336. PubMed PMID: 26394555.
23. Malhi GS, Tanious M, Das P, Coulston CM, Berk M. Potential mechanisms of action of lithium in bipolar disorder. Current understanding. CNS Drugs. 2013;27(2):135-53. doi: 10.1007/s40263-013-0039-0. PubMed PMID: 23371914.
24. Usher J, Menzel P, Schneider-Axmann T, Kemmer C, Reith W, Falkai P, Gruber O, Scherk H. Increased right amygdala volume in lithium-treated patients with bipolar I disorder. Acta psychiatrica Scandinavica. 2010;121(2):119-24. doi: 10.1111/j.1600-0447.2009.01428.x. PubMed PMID: 19573050.
25. Foland LC, Altshuler LL, Sugar CA, Lee AD, Leow AD, Townsend J, Narr KL, Asuncion DM, Toga AW, Thompson PM. Increased volume of the amygdala and hippocampus in bipolar patients treated with lithium. Neuroreport. 2008;19(2):221-4. Epub 2008/01/11. doi: 10.1097/WNR.0b013e3282f48108 00001756-200801220-00017 [pii]. PubMed PMID: 18185112.
26. Hajek T, Cullis J, Novak T, Kopecek M, Hoschl C, Blagdon R, O'Donovan C, Bauer M, Young LT, Macqueen G, Alda M. Hippocampal volumes in bipolar disorders: opposing effects of illness burden and lithium treatment. Bipolar Disord. 2012;14(3):261-70. Epub 2012/05/03. doi: 10.1111/j.1399-5618.2012.01013.x. PubMed PMID: 22548899.
27. Hajek T, Kopecek M, Hoschl C, Alda M. Smaller hippocampal volumes in patients with bipolar disorder are masked by exposure to lithium: a meta-analysis. J Psychiatry Neurosci. 2012;37(3):110143. Epub 2012/04/14. doi: 10.1503/jpn.110143 10.1503/cjs.110143 [pii]. PubMed PMID: 22498078.
28. Macritchie KA, Lloyd AJ, Bastin ME, Vasudev K, Gallagher P, Eyre R, Marshall I, Wardlaw JM, Ferrier IN, Moore PB, Young AH. White matter microstructural abnormalities in euthymic bipolar disorder. The British journal of psychiatry : the journal of mental science. 2010;196(1):52-8. Epub 2010/01/02. doi: 196/1/52 [pii] 10.1192/bjp.bp.108.058586. PubMed PMID: 20044661.
29. Schloesser RJ, Huang J, Klein PS, Manji HK. Cellular plasticity cascades in the pathophysiology and treatment of bipolar disorder. Neuropsychopharmacology : official publication of the American College of Neuropsychopharmacology. 2008;33(1):110-33. Epub 2007/10/04. doi: 1301575 [pii] 10.1038/sj.npp.1301575. PubMed PMID: 17912251.
30. Forlenza OV, Aprahamian I, de Paula VJ, Hajek T. Lithium, a therapy for AD: current evidence from clinical trials of neurodegenerative disorders. Current Alzheimer research. 2016. PubMed PMID: 26892289.
31. Forlenza OV, de Paula VJ, Machado-Vieira R, Diniz BS, Gattaz WF. Does lithium prevent Alzheimer's disease? Drugs & aging. 2012;29(5):335-42. doi: 10.2165/11599180-000000000-00000. PubMed PMID: 22500970.
32. Quiroz JA, Gould TD, Manji HK. Molecular effects of lithium. Mol Interv. 2004;4(5):259-72. Epub 2004/10/09. doi: 4/5/259 [pii] 10.1124/mi.4.5.6. PubMed PMID: 15471909.
33. Moore GJ, Bebchuk JM, Hasanat K, Chen G, Seraji-Bozorgzad N, Wilds IB, Faulk MW, Koch S, Glitz DA, Jolkovsky L, Manji HK. Lithium increases N-acetyl-aspartate in the human brain: in vivo evidence in support of bcl-2's neurotrophic effects? Biol Psychiatry. 2000;48(1):1-8. Epub 2000/07/29. doi: S0006-3223(00)00252-3 [pii]. PubMed PMID: 10913502.
34. Moore GJ, Bebchuk JM, Wilds IB, Chen G, Manji HK. Lithium-induced increase in human brain grey matter. Lancet. 2000;356(9237):1241-2. Epub 2000/11/10. doi: S0140673600027938 [pii]. PubMed PMID: 11072948.
35. Bachmann RF, Wang Y, Yuan P, Zhou R, Li X, Alesci S, Du J, Manji HK. Common effects of lithium and valproate on mitochondrial functions: protection against methamphetamine-induced mitochondrial damage. The international journal of neuropsychopharmacology / official scientific journal of the Collegium Internationale Neuropsychopharmacologicum. 2009;12(6):805-22. Epub 2009/01/20. doi: S1461145708009802 [pii] 10.1017/S1461145708009802. PubMed PMID: 19149911; PubMed Central PMCID: PMC2779114.

36. Chen G, Zeng WZ, Yuan PX, Huang LD, Jiang YM, Zhao ZH, Manji HK. The mood-stabilizing agents lithium and valproate robustly increase the levels of the neuroprotective protein bcl-2 in the CNS. Journal of neurochemistry. 1999;72(2):879-82. Epub 1999/02/04. PubMed PMID: 9930766.
37. Machado-Vieira R, Soeiro-De-Souza MG, Richards EM, Teixeira AL, Zarate CA, Jr. Multiple levels of impaired neural plasticity and cellular resilience in bipolar disorder: developing treatments using an integrated translational approach. The world journal of biological psychiatry : the official journal of the World Federation of Societies of Biological Psychiatry. 2014;15(2):84-95. doi: 10.3109/15622975.2013.830775. PubMed PMID: 23998912; PubMed Central PMCID: PMC4180367.
38. Berk M, Conus P, Kapczinski F, Andreazza AC, Yucel M, Wood SJ, Pantelis C, Malhi GS, Dodd S, Bechdolf A, Amminger GP, Hickie IB, McGorry PD. From neuroprogression to neuroprotection: implications for clinical care. Med J Aust. 2010;193(4 Suppl):S36-40. Epub 2010/08/18. doi: ber10227_fm [pii]. PubMed PMID: 20712560.
39. Young W. Review of lithium effects on brain and blood. Cell Transplant. 2009;18(9):951-75. Epub 2009/06/16. doi: CT-2065 [pii] 10.3727/096368909X471251. PubMed PMID: 19523343.
40. Khairova R, Pawar R, Salvadore G, Juruena MF, de Sousa RT, Soeiro-de-Souza MG, Salvador M, Zarate CA, Gattaz WF, Machado-Vieira R. Effects of lithium on oxidative stress parameters in healthy subjects. Mol Med Report. 2012;5(3):680-2. doi: 10.3892/mmr.2011.732. PubMed PMID: 22200861; PubMed Central PMCID: PMC3289682.
41. Gray JD, McEwen BS. Lithium's role in neural plasticity and its implications for mood disorders. Acta psychiatrica Scandinavica. 2013;128(5):347-61. doi: 10.1111/acps.12139. PubMed PMID: 23617566; PubMed Central PMCID: PMCPMC3743945.
42. Forlenza OV, Diniz BS, Radanovic M, Santos FS, Talib LL, Gattaz WF. Disease-modifying properties of long-term lithium treatment for amnestic mild cognitive impairment: randomised controlled trial. The British journal of psychiatry : the journal of mental science. 2011;198:351-6. Epub 2011/04/29. doi: 198/5/351 [pii] 10.1192/bjp.bp.110.080044. PubMed PMID: 21525519.
43. Leyhe T, Eschweiler GW, Stransky E, Gasser T, Annas P, Basun H, Laske C. Increase of BDNF serum concentration in lithium treated patients with early Alzheimer's disease. Journal of Alzheimer's disease : JAD. 2009;16(3):649-56. doi: 10.3233/JAD-2009-1004. PubMed PMID: 19276559.
44. Dunn N, Holmes C, Mullee M. Does lithium therapy protect against the onset of dementia? Alzheimer disease and associated disorders. 2005;19(1):20-2. PubMed PMID: 15764867.
45. da Silva J, Gonçalves-Pereira M, Xavier M, Mukaetova-Ladinska EB. Affective disorders and risk of developing dementia: systematic review. The British Journal of Psychiatry. 2013;202(3):177-86. doi: 10.1192/bjp.bp.111.101931.
46. Kerchner GA. Ultra-high field 7T MRI: a new tool for studying Alzheimer's disease. Journal of Alzheimer's disease : JAD. 2011;26 Suppl 3:91-5. doi: 10.3233/JAD-2011-0023. PubMed PMID: 21971453.
47. van der Kolk AG, Hendrikse J, Zwanenburg JJ, Visser F, Luijten PR. Clinical applications of 7 T MRI in the brain. Eur J Radiol. 2013;82(5):708-18. doi: 10.1016/j.ejrad.2011.07.007. PubMed PMID: 21937178.
48. Lyoo IK, Dager SR, Kim JE, Yoon SJ, Friedman SD, Dunner DL, Renshaw PF. Lithium-induced gray matter volume increase as a neural correlate of treatment response in bipolar disorder: a longitudinal brain imaging study. Neuropsychopharmacology : official publication of the American College of Neuropsychopharmacology. 2010;35(8):1743-50. doi: 10.1038/npp.2010.41. PubMed PMID: 20357761; PubMed Central PMCID: PMC3055479.
49. Yucel K, Taylor VH, McKinnon MC, Macdonald K, Alda M, Young LT, MacQueen GM. Bilateral hippocampal volume increase in patients with bipolar disorder and short-term lithium treatment. Neuropsychopharmacology : official publication of the American College of Neuropsychopharmacology. 2008;33(2):361-7. doi: 10.1038/sj.npp.1301405. PubMed PMID: 17406649.
50. Monkul ES, Matsuo K, Nicoletti MA, Dierschke N, Hatch JP, Dalwani M, Brambilla P, Caetano S, Sassi RB, Mallinger AG, Soares JC. Prefrontal gray matter increases in healthy individuals after lithium treatment: a voxel-based morphometry study. Neuroscience letters. 2007;429(1):7-11. doi: 10.1016/j.neulet.2007.09.074. PubMed PMID: 17996370; PubMed Central PMCID: PMC2693231.
51. Moore GJ, Cortese BM, Glitz DA, Zajac-Benitez C, Quiroz JA, Uhde TW, Drevets WC, Manji HK. A longitudinal study of the effects of lithium treatment on prefrontal and subgenual prefrontal gray matter volume in treatment-responsive bipolar disorder patients. The Journal of clinical psychiatry. 2009;70(5):699-705. doi: 10.4088/JCP.07m03745. PubMed PMID: 19389332.
52. Gildengers AG, Chisholm D, Butters MA, Anderson SJ, Begley A, Holm M, Rogers JC, Reynolds CF, Mulsant BH. Two-year course of cognitive function and instrumental activities of daily living in older adults

with bipolar disorder: evidence for neuroprogression? Psychological medicine. 2012:1-11. doi: 10.1017/S0033291712001614. PubMed PMID: 22846332.
53. Gildengers AG, Mulsant BH, Begley A, Mazumdar S, Hyams AV, Reynolds Iii CF, Kupfer DJ, Butters MA. The longitudinal course of cognition in older adults with bipolar disorder. Bipolar Disord. 2009;11(7):744-52. Epub 2009/09/02. doi: BDI739 [pii] 10.1111/j.1399-5618.2009.00739.x. PubMed PMID: 19719787; PubMed Central PMCID: PMC2887714.
54. Reynolds CF, 3rd, Dew MA, Pollock BG, Mulsant BH, Frank E, Miller MD, Houck PR, Mazumdar S, Butters MA, Stack JA, Schlernitzauer MA, Whyte EM, Gildengers A, Karp J, Lenze E, Szanto K, Bensasi S, Kupfer DJ. Maintenance treatment of major depression in old age. The New England journal of medicine. 2006;354(11):1130-8. Epub 2006/03/17. doi: 354/11/1130 [pii] 10.1056/NEJMoa052619. PubMed PMID: 16540613.
55. Reynolds CF, 3rd, Butters MA, Lopez O, Pollock BG, Dew MA, Mulsant BH, Lenze EJ, Holm M, Rogers JC, Mazumdar S, Houck PR, Begley A, Anderson S, Karp JF, Miller MD, Whyte EM, Stack J, Gildengers A, Szanto K, Bensasi S, Kaufer DI, Kamboh MI, DeKosky ST. Maintenance treatment of depression in old age: a randomized, double-blind, placebo-controlled evaluation of the efficacy and safety of donepezil combined with antidepressant pharmacotherapy. Archives of general psychiatry. 2011;68(1):51-60. Epub 2011/01/05. doi: 68/1/51 [pii] 10.1001/archgenpsychiatry.2010.184. PubMed PMID: 21199965; PubMed Central PMCID: PMC3076045.
56. Butters MA, Whyte EM, Nebes RD, Begley AE, Dew MA, Mulsant BH, Zmuda MD, Bhalla R, Meltzer CC, Pollock BG, Reynolds CF, III, Becker JT. The Nature and Determinants of Neuropsychological Functioning in Late-Life Depression. Archives of general psychiatry. 2004;61(6):587-95. doi: 10.1001/archpsyc.61.6.587.
57. Fagiolini A, Frank E, Axelson DA, Birmaher B, Cheng Y, Curet DE, Friedman ES, Gildengers AG, Goldstein T, Grochocinski VJ, Houck PR, Stofko MG, Thase ME, Thompson WK, Turkin SR, Kupfer DJ. Enhancing outcomes in patients with bipolar disorder: results from the Bipolar Disorder Center for Pennsylvanians Study. Bipolar Disord. 2009;11(4):382-90. Epub 2009/06/09. doi: BDI700 [pii] 10.1111/j.1399-5618.2009.00700.x. PubMed PMID: 19500091; PubMed Central PMCID: PMC3361715.
58. Gildengers AG, Butters MA, Chisholm D, Reynolds CF, Mulsant BH. A 12-week open-label pilot study of donepezil for cognitive functioning and instrumental activities of daily living in late-life bipolar disorder. International journal of geriatric psychiatry. 2008;23(7):693-8. Epub 2008/01/04. doi: 10.1002/gps.1962. PubMed PMID: 18172910; PubMed Central PMCID: PMC2771198.
59. Gildengers AG, Mulsant BH, Begley AE, McShea M, Stack JA, Miller MD, Fagiolini A, Kupfer DJ, Young RC, Reynolds CF, 3rd. A pilot study of standardized treatment in geriatric bipolar disorder. The American journal of geriatric psychiatry : official journal of the American Association for Geriatric Psychiatry. 2005;13(4):319-23. Epub 2005/04/23. doi: 13/4/319 [pii] 10.1176/appi.ajgp.13.4.319. PubMed PMID: 15845758.
60. Sajatovic M, Gildengers A, Al Jurdi RK, Gyulai L, Cassidy KA, Greenberg RL, Bruce ML, Mulsant BH, Ten Have T, Young RC. Multisite, open-label, prospective trial of lamotrigine for geriatric bipolar depression: a preliminary report. Bipolar Disord. 2011;13(3):294-302. Epub 2011/06/17. doi: 10.1111/j.1399-5618.2011.00923.x. PubMed PMID: 21676132.
61. Young RC, Schulberg HC, Gildengers AG, Sajatovic M, Mulsant BH, Gyulai L, Beyer J, Marangell L, Kunik M, Ten Have T, Bruce ML, Gur R, Marino P, Evans JD, Reynolds CF, 3rd, Alexopoulos GS. Conceptual and methodological issues in designing a randomized, controlled treatment trial for geriatric bipolar disorder: GERI-BD. Bipolar Disord. 2010;12(1):56-67. Epub 2010/02/13. doi: BDI779 [pii] 10.1111/j.1399-5618.2009.00779.x. PubMed PMID: 20148867; PubMed Central PMCID: PMC3039416.
62. Gildengers AG, Butters MA, Aizenstein HJ, Marron MM, Emanuel J, Anderson SJ, Weissfeld LA, Becker JT, Lopez OL, Mulsant BH, Reynolds CF. Longer lithium exposure is associated with better white matter integrity in older adults with bipolar disorder. Bipolar Disorders. 2015;17(3):248-56. doi: DOI 10.1111/bdi.12260. PubMed PMID: WOS:000353402800002.
63. Gildengers AG, Butters MA, Albert SM, Anderson SJ, Dew MA, Erickson K, Garand L, Karp JF, Lockovich MH, Morse J, Reynolds CF, 3rd. Design and Implementation of an Intervention Development Study: Retaining Cognition While Avoiding Late-Life Depression (ReCALL). The American journal of geriatric psychiatry : official journal of the American Association for Geriatric Psychiatry. 2015. doi: 10.1016/j.jagp.2015.10.010. PubMed PMID: 27066730.
64. Diniz BS, Sibille E, Ding Y, Tseng G, Aizenstein HJ, Lotrich F, Becker JT, Lopez OL, Lotze MT, Klunk WE, Reynolds CF, Butters MA. Plasma biosignature and brain pathology related to persistent cognitive

impairment in late-life depression. Molecular psychiatry. 2015;20(5):594-601. doi: 10.1038/mp.2014.76. PubMed PMID: 25092249; PubMed Central PMCID: PMCPMC4494754.

65. Manji HK, Duman RS. Impairments of neuroplasticity and cellular resilience in severe mood disorders: implications for the development of novel therapeutics. Psychopharmacol Bull. 2001;35(2):5-49. PubMed PMID: 12397885.
66. Anderson K, Jue SG, Madaras-Kelly KJ. Identifying patients at risk for medication mismanagement: using cognitive screens to predict a patient's accuracy in filling a pillbox. Consult Pharm. 2008;23(6):459-72. PubMed PMID: 18764676.
67. Lam AY, Anderson K, Borson S, Smith FL. A pilot study to assess cognition and pillbox fill accuracy by community-dwelling older adults. Consult Pharm. 2011;26(4):256-63. doi: 10.4140/TCP.n.2011.256. PubMed PMID: 21486736.
68. Delis DC, Kramer JH, Kaplan E, Ober BA. California Verbal Learning Test Manual. New York, NY: Psychological Corporation; 1987.
69. Benedict RH, Schretlen D, Groninger L, Dobraski M, Shpritz B. Revision of the Brief Visuospatial Memory Test: Studies of normal performance, reliability, and validity. Psychological Assessment. 1996;8(2):145-53. PubMed PMID: 1996-00455-005.
70. Teng EL, Chui HC. The Modified Mini-Mental State (3MS) examination. The Journal of clinical psychiatry. 1987;48(8):314-8. PubMed PMID: 3611032.
71. Reitan RM. Validity of the Trail Making test as an indicator of organic brain damage. Perceptual and motor skills. 1958;8:271-6.
72. O'Caoimh R, Gao Y, McGlade C, Healy L, Gallagher P, Timmons S, Molloy DW. Comparison of the quick mild cognitive impairment (Qmci) screen and the SMMSE in screening for mild cognitive impairment. Age and ageing. 2012;41(5):624-9. doi: 10.1093/ageing/afs059. PubMed PMID: 22610464; PubMed Central PMCID: PMC3424052.
73. Sheehan DV, Lecrubier Y, Sheehan KH, Amorim P, Janavs J, Weiller E, Hergueta T, Baker R, Dunbar GC. The Mini-International Neuropsychiatric Interview (M.I.N.I.): the development and validation of a structured diagnostic psychiatric interview for DSM-IV and ICD-10. The Journal of clinical psychiatry. 1998;59 Suppl 20:22-33;quiz 4-57. PubMed PMID: 9881538.
74. Morris JC. The Clinical Dementia Rating (CDR): current version and scoring rules. Neurology. 1993;43(11):2412-4. PubMed PMID: 8232972.
75. O'Bryant SE, Lacritz LH, Hall J, Waring SC, Chan W, Khodr ZG, Massman PJ, Hobson V, Cullum CM. Validation of the new interpretive guidelines for the clinical dementia rating scale sum of boxes score in the national Alzheimer's coordinating center database. Archives of neurology. 2010;67(6):746-9. doi: 10.1001/archneurol.2010.115. PubMed PMID: 20558394; PubMed Central PMCID: PMCPMC2888493.
76. Farias ST, Mungas D, Reed BR, Cahn-Weiner D, Jagust W, Baynes K, Decarli C. The measurement of everyday cognition (ECog): scale development and psychometric properties. Neuropsychology. 2008;22(4):531-44. doi: 10.1037/0894-4105.22.4.531. PubMed PMID: 18590364; PubMed Central PMCID: PMC2877034.
77. Movement Disorder Society Task Force on Rating Scales for Parkinson's D. The Unified Parkinson's Disease Rating Scale (UPDRS): status and recommendations. Movement disorders : official journal of the Movement Disorder Society. 2003;18(7):738-50. doi: 10.1002/mds.10473. PubMed PMID: 12815652.
78. Wilkinson GS, Robertson GJ. Wide range achievement test – Fourth edition: Professional manual. Lutz, FL: Psychological Assessment Resources; 2006.
79. Wilk CM, Gold JM, Bartko JJ, Dickerson F, Fenton WS, Knable M, Randolph C, Buchanan RW. Test-Retest Stability of the Repeatable Battery for the Assessment of Neuropsychological Status in Schizophrenia. The American journal of psychiatry. 2002;159(5):838-44.
80. Delis DC, Kaplan E, Kramer JH. Delis-Kaplan Executive Function System. San Antonio, TX: The Psychological Corporation, a Harcourt Assessment Company; 2001.
81. Chisholm D, Toto P, Raina K, Holm M, Rogers J. Evaluating capacity to live independently and safely in the community: Performance Assessment of Self-care Skills. Br J Occup Ther. 2014;77(2):59-63. doi: 10.4276/030802214X13916969447038. PubMed PMID: 25298616; PubMed Central PMCID: PMCPMC4186770.
82. Rodakowski J, Skidmore ER, Reynolds CF, 3rd, Dew MA, Butters MA, Holm MB, Lopez OL, Rogers JC. Can performance on daily activities discriminate between older adults with normal cognitive function and those with mild cognitive impairment? Journal of the American Geriatrics Society. 2014;62(7):1347-52. doi: 10.1111/jgs.12878. PubMed PMID: 24890517; PubMed Central PMCID: PMCPMC4107156.

83. Kroenke K, Spitzer RL, Williams JB. The PHQ-9: validity of a brief depression severity measure. Journal of general internal medicine. 2001;16(9):606-13. PubMed PMID: 11556941; PubMed Central PMCID: PMCPMC1495268.
84. Miller MD, Paradis CF, Houck PR, Mazumdar S, Stack JA, Rifai AH, Mulsant B, Reynolds CF, 3rd. Rating chronic medical illness burden in geropsychiatric practice and research: application of the Cumulative Illness Rating Scale. Psychiatry research. 1992;41(3):237-48.
85. Wolf PA, D'Agostino RB, Belanger AJ, Kannel WB. Probability of stroke: a risk profile from the Framingham Study. Stroke; a journal of cerebral circulation. 1991;22(3):312-8.
86. Byerly MJ, Nakonezny PA, Rush AJ. The Brief Adherence Rating Scale (BARS) validated against electronic monitoring in assessing the antipsychotic medication adherence of outpatients with schizophrenia and schizoaffective disorder. Schizophrenia research. 2008;100(1-3):60-9. doi: 10.1016/j.schres.2007.12.470. PubMed PMID: 18255269.
87. Lingjaerde O, Ahlfors UG, Bech P, Dencker SJ, Elgen K. The UKU side effect rating scale. A new comprehensive rating scale for psychotropic drugs and a cross-sectional study of side effects in neuroleptic-treated patients. Acta Psychiatrica Scandinavica, Supplementum. 1987;334:1-100.
88. Washburn RA, Smith KW, Jette AM, Janney CA. The Physical Activity Scale for the Elderly (PASE): development and evaluation. Journal of clinical epidemiology. 1993;46(2):153-62. PubMed PMID: 8437031.
89. Lopez OL, Kuller LH, Fitzpatrick A, Ives D, Becker JT, Beauchamp N. Evaluation of dementia in the cardiovascular health cognition study. Neuroepidemiology. 2003;22(1):1-12. Epub 2003/02/05. doi: 10.1159/000067110 ned22001 [pii]. PubMed PMID: 12566948.
90. McKhann GM, Knopman DS, Chertkow H, Hyman BT, Jack CR, Jr., Kawas CH, Klunk WE, Koroshetz WJ, Manly JJ, Mayeux R, Mohs RC, Morris JC, Rossor MN, Scheltens P, Carrillo MC, Thies B, Weintraub S, Phelps CH. The diagnosis of dementia due to Alzheimer's disease: recommendations from the National Institute on Aging-Alzheimer's Association workgroups on diagnostic guidelines for Alzheimer's disease. Alzheimer's & dementia : the journal of the Alzheimer's Association. 2011;7(3):263-9. doi: 10.1016/j.jalz.2011.03.005. PubMed PMID: 21514250; PubMed Central PMCID: PMC3312024.
91. Petersen RC. Mild cognitive impairment as a diagnostic entity. Journal of internal medicine. 2004;256(3):183-94. doi: 10.1111/j.1365-2796.2004.01388.x. PubMed PMID: 15324362.
92. Forlenza OV, Torres CA, Talib LL, de Paula VJ, Joaquim HP, Diniz BS, Gattaz WF. Increased platelet GSK3B activity in patients with mild cognitive impairment and Alzheimer's disease. Journal of psychiatric research. 2011;45(2):220-4. doi: 10.1016/j.jpsychires.2010.06.002. PubMed PMID: 20576277.
93. Dou H, Ellison B, Bradley J, Kasiyanov A, Poluektova LY, Xiong H, Maggirwar S, Dewhurst S, Gelbard HA, Gendelman HE. Neuroprotective mechanisms of lithium in murine human immunodeficiency virus-1 encephalitis. The Journal of neuroscience : the official journal of the Society for Neuroscience. 2005;25(37):8375-85. doi: 10.1523/JNEUROSCI.2164-05.2005. PubMed PMID: 16162919.
94. Lotrich FE, Butters MA, Aizenstein H, Marron MM, Reynolds CF, 3rd, Gildengers AG. The relationship between interleukin-1 receptor antagonist and cognitive function in older adults with bipolar disorder. International journal of geriatric psychiatry. 2014;29(6):635-44. doi: 10.1002/gps.4048. PubMed PMID: 24273017; PubMed Central PMCID: PMC4013203.
95. Kitazawa M, Trinh DN, LaFerla FM. Inflammation induces tau pathology in inclusion body myositis model via glycogen synthase kinase-3beta. Annals of neurology. 2008;64(1):15-24. doi: 10.1002/ana.21325. PubMed PMID: 18318434.
96. Chew ML, Mulsant BH, Pollock BG, Lehman ME, Greenspan A, Mahmoud RA, Kirshner MA, Sorisio DA, Bies RR, Gharabawi G. Anticholinergic Activity of 107 Medications Commonly Used by Older Adults. Journal of the American Geriatrics Society. 2008. Epub 2008/05/31. doi: JGS1737 [pii] 10.1111/j.1532-5415.2008.01737.x. PubMed PMID: 18510583.
97. Nebes RD, Pollock BG, Mulsant BH, Kirshner MA, Halligan E, Zmuda M, Reynolds CF, 3rd. Low-level serum anticholinergicity as a source of baseline cognitive heterogeneity in geriatric depressed patients. Psychopharmacology Bulletin. 1997;33(4):715-20.
98. Martinsson L, Wei Y, Xu D, Melas PA, Mathe AA, Schalling M, Lavebratt C, Backlund L. Long-term lithium treatment in bipolar disorder is associated with longer leukocyte telomeres. Translational psychiatry. 2013;3:e261. doi: 10.1038/tp.2013.37. PubMed PMID: 23695236; PubMed Central PMCID: PMC3669924.
99. Prather AA, Rabinovitz M, Pollock BG, Lotrich FE. Cytokine-induced depression during IFN-alpha treatment: the role of IL-6 and sleep quality. Brain, behavior, and immunity. 2009;23(8):1109-16. doi: 10.1016/j.bbi.2009.07.001. PubMed PMID: 19615438; PubMed Central PMCID: PMC2783448.

100. Soman S, Holdsworth SJ, Barnes PD, Rosenberg J, Andre JB, Bammer R, Yeom KW. Improved T2* imaging without increase in scan time: SWI processing of 2D gradient echo. Ajnr. 2013;34(11):2092-7. Epub 2013/06/08. doi: 10.3174/ajnr.A3595. PubMed PMID: 23744690.
101. Yushkevich PA, Amaral RS, Augustinack JC, Bender AR, Bernstein JD, Boccardi M, Bocchetta M, Burggren AC, Carr VA, Chakravarty MM, Chetelat G, Daugherty AM, Davachi L, Ding SL, Ekstrom A, Geerlings MI, Hassan A, Huang Y, Iglesias JE, La Joie R, Kerchner GA, LaRocque KF, Libby LA, Malykhin N, Mueller SG, Olsen RK, Palombo DJ, Parekh MB, Pluta JB, Preston AR, Pruessner JC, Ranganath C, Raz N, Schlichting ML, Schoemaker D, Singh S, Stark CE, Suthana N, Tompary A, Turowski MM, Van Leemput K, Wagner AD, Wang L, Winterburn JL, Wisse LE, Yassa MA, Zeineh MM, Hippocampal Subfields G. Quantitative comparison of 21 protocols for labeling hippocampal subfields and parahippocampal subregions in in vivo MRI: Towards a harmonized segmentation protocol. NeuroImage. 2015;111:526-41. doi: 10.1016/j.neuroimage.2015.01.004. PubMed PMID: 25596463; PubMed Central PMCID: PMC4387011.
102. Convit A, McHugh P, Wolf OT, de Leon MJ, Bobinski M, De Santi S, Roche A, Tsui W. MRI volume of the amygdala: a reliable method allowing separation from the hippocampal formation. Psychiatry research. 1999;90(2):113-23. PubMed PMID: 10482383.
103. Entis JJ, Doerga P, Barrett LF, Dickerson BC. A reliable protocol for the manual segmentation of the human amygdala and its subregions using ultra-high resolution MRI. NeuroImage. 2012;60(2):1226-35. doi: 10.1016/j.neuroimage.2011.12.073. PubMed PMID: 22245260; PubMed Central PMCID: PMC3665767.
104. Rej S, Butters MA, Aizenstein HJ, Begley A, Tsay J, Reynolds CF, 3rd, Mulsant BH, Gildengers A. Neuroimaging and neurocognitive abnormalities associated with bipolar disorder in old age. International journal of geriatric psychiatry. 2013. doi: 10.1002/gps.4021. PubMed PMID: 24006234.
105. Holmes CJ, Hoge R, Collins L, Woods R, Toga AW, Evans AC. Enhancement of MR images using registration for signal averaging. J Comput Assist Tomogr. 1998;22(2):324-33. Epub 1998/04/08. PubMed PMID: 9530404.
106. Freesurfer. Available from: www.surfer.nmr.mgh.harvard.edu.
107. Greenberg SM, Vernooij MW, Cordonnier C, Viswanathan A, Al-Shahi Salman R, Warach S, Launer LJ, Van Buchem MA, Breteler MM, Microbleed Study G. Cerebral microbleeds: a guide to detection and interpretation. The Lancet Neurology. 2009;8(2):165-74. doi: 10.1016/S1474-4422(09)70013-4. PubMed PMID: 19161908; PubMed Central PMCID: PMC3414436.
108. Novelli EM, Elizabeth Sarles C, Jay Aizenstein H, Ibrahim TS, Butters MA, Connelly Ritter A, Erickson KI, Rosano C. Brain venular pattern by 7T MRI correlates with memory and haemoglobin in sickle cell anaemia. Psychiatry research. 2015;233(1):18-22. doi: 10.1016/j.pscychresns.2015.04.005. PubMed PMID: 26002434.
109. Wu M, Rosano C, Butters M, Whyte E, Nable M, Crooks R, Meltzer CC, Reynolds CF, 3rd, Aizenstein HJ. A fully automated method for quantifying and localizing white matter hyperintensities on MR images. Psychiatry research. 2006;148(2-3):133-42. Epub 2006/11/14. doi: S0925-4927(06)00133-8 [pii] 10.1016/j.pscychresns.2006.09.003. PubMed PMID: 17097277.
110. Wu M, Rosano C, Lopez-Garcia P, Carter CS, Aizenstein HJ. Optimum template selection for atlas-based segmentation. NeuroImage. 2007;34(4):1612-8. Epub 2006/12/26. doi: S1053-8119(06)00809-3 [pii] 10.1016/j.neuroimage.2006.07.050. PubMed PMID: 17188896.
111. Mori S, Crain BJ. MRI atlas of human white matter. 1st ed. Amsterdam; Boston: Elsevier; 2005. viii, p. 239
112. Venkatraman VK, Aizenstein HJ, Newman AB, Yaffe K, Harris T, Kritchevsky S, Ayonayon HN, Rosano C. Lower Digit Symbol Substitution Score in the Oldest Old is Related to Magnetization Transfer and Diffusion Tensor Imaging of the White Matter. Frontiers in aging neuroscience. 2011;3:11. doi: 10.3389/fnagi.2011.00011. PubMed PMID: 21991255; PubMed Central PMCID: PMC3180637.
113. Smith SM, Jenkinson M, Johansen-Berg H, Rueckert D, Nichols TE, Mackay CE, Watkins KE, Ciccarelli O, Cader MZ, Matthews PM, Behrens TE. Tract-based spatial statistics: voxelwise analysis of multi-subject diffusion data. NeuroImage. 2006;31(4):1487-505. Epub 2006/04/21. doi: S1053-8119(06)00138-8 [pii] 10.1016/j.neuroimage.2006.02.024. PubMed PMID: 16624579.
114. Karim HT, Andreescu C, Tudorascu D, Smagula SF, Butters MA, Karp JF, Reynolds C, Aizenstein HJ. Intrinsic functional connectivity in late-life depression: trajectories over the course of pharmacotherapy in remitters and non-remitters. Molecular psychiatry. 2016. doi: 10.1038/mp.2016.55. PubMed PMID: 27090303.
115. Lim HK, Nebes R, Snitz B, Cohen A, Mathis C, Price J, Weissfeld L, Klunk W, Aizenstein HJ. Regional amyloid burden and intrinsic connectivity networks in cognitively normal elderly subjects. Brain : a journal

of neurology. 2014;137(Pt 12):3327-38. doi: 10.1093/brain/awu271. PubMed PMID: 25266592; PubMed Central PMCID: PMCPMC4240287.
116. Karim HK, Andreescu C, MacCloud R, Butters MA, Reynolds C, Aizenstein HJ, Tudorascu DL. The effects of white matter disease on the accuracy of automated segmentation. Psychiatry Research: Neuroimaging. In Press.
117. Shirer WR, Ryali S, Rykhlevskaia E, Menon V, Greicius MD. Decoding subject-driven cognitive states with whole-brain connectivity patterns. Cerebral cortex. 2012;22(1):158-65. doi: 10.1093/cercor/bhr099. PubMed PMID: 21616982; PubMed Central PMCID: PMCPMC3236795.
118. Mathis C, Wang Y, Holt D, Huang G-F, Debnath M, Klunk W. Synthesis and evaluation of ^{11}C-labeled 6-substituted 2-aryl benzothiazoles as amyloid imaging agents. J Med Chem. 2003;46:2740-55.
119. Klunk W, Engler H, Nordberg A, Wang Y, Blomqvist G, DP DH, Bergstrom M, Savitcheva I, Huang G-F, Estrada S, Ausen B, Debnath M, Barletta J, Price J, Sandell J, Lopresti B, Wall A, Koivisto P, Antoni G, Mathis C, Langstrom B. Imaging Brain Amyloid in Alzheimer's Disease Using the Novel PET Tracer PIB. Annals of Neurology. 2004;55:306-19.
120. Wilson A, A,, A. G, Chestakova A, Kung HF, Houle S. A rapid one-step radiosynthesis of the beta-amyloid imaging radiotracer N-methyl-[C-11]2-(4'-methylaminophenyl)-6-hydroxybenzothiazole ([C-11]-6-OHBTA-1. J Labelled Compounds and Radiopharmaceuticals. 2004;47:679-82.
121. PMOD. Available from: http://www.pmod.com.
122. Rousset OG, Collins DL, Rahmim A, Wong DF. Design and implementation of an automated partial volume correction in PET: application to dopamine receptor quantification in the normal human striatum. J Nucl Med. 2008;49(7):1097-106. PubMed PMID: 18552147.
123. Rousset OG, Ma Y, Evans AC. Correction for partial volume effects in PET: principle and validation. J Nucl Med. 1998;39(5):904-11. PubMed PMID: 9591599.
124. Cohen SI, Linse S, Luheshi LM, Hellstrand E, White DA, Rajah L, Otzen DE, Vendruscolo M, Dobson CM, Knowles TP. Proliferation of amyloid-beta42 aggregates occurs through a secondary nucleation mechanism. Proceedings of the National Academy of Sciences of the United States of America. 2013;110(24):9758-63. Epub 2013/05/25. doi: 10.1073/pnas.1218402110. PubMed PMID: 23703910; PubMed Central PMCID: PMC3683769.
125. Klunk WE, Price JC, Mathis CA, Tsopelas ND, Lopresti BJ, Ziolko SK, Bi W, Hoge JA, Cohen AD, Ikonomovic MD, Saxton JA, Snitz BE, Pollen DA, Moonis M, Lippa CF, Swearer JM, Johnson KA, Rentz DM, Fischman AJ, Aizenstein HJ, DeKosky ST. Amyloid deposition begins in the striatum of presenilin-1 mutation carriers from two unrelated pedigrees. J Neurosci. 2007;27(23):6174-84. PubMed PMID: 17553989.
126. ICBM 152 atlas. Available from: http://www.bic.mni.mcgill.ca/ServicesAtlases.
127. Association AP. Practice Guidelines for the Treatment of Psychiatric Disorders. Washington, DC: American Psychiatric Association; 2002.
128. Yatham LN, Kennedy SH, O'Donovan C, Parikh S, MacQueen G, McIntyre R, Sharma V, Silverstone P, Alda M, Baruch P, Beaulieu S, Daigneault A, Milev R, Young LT, Ravindran A, Schaffer A, Connolly M, Gorman CP. Canadian Network for Mood and Anxiety Treatments (CANMAT) guidelines for the management of patients with bipolar disorder: consensus and controversies. Bipolar Disorders. 2005;7(s3):5-69. doi: doi:10.1111/j.1399-5618.2005.00219.x.
129. Yatham LN, Kennedy SH, O'Donovan C, Parikh SV, MacQueen G, McIntyre RS, Sharma V, Beaulieu S. Canadian Network for Mood and Anxiety Treatments (CANMAT) guidelines for the management of patients with bipolar disorder: update 2007. Bipolar Disorders. 2006;8(6):721-39. doi: doi:10.1111/j.1399-5618.2006.00432.x.
130. Yatham LN, Kennedy SH, Schaffer A, Parikh SV, Beaulieu S, O'Donovan C, MacQueen G, McIntyre RS, Sharma V, Ravindran A, Young LT, Young AH, Alda M, Milev R, Vieta E, Calabrese JR, Berk M, Ha K, Kapczinski F. Canadian Network for Mood and Anxiety Treatments (CANMAT) and International Society for Bipolar Disorders (ISBD) collaborative update of CANMAT guidelines for the management of patients with bipolar disorder: update 2009. Bipolar Disord. 2009;11(3):225-55. Epub 2009/05/08. doi: BDI672 [pii] 10.1111/j.1399-5618.2009.00672.x. PubMed PMID: 19419382.
131. Young RC, Gyulai L, Mulsant BH, Flint A, Beyer JL, Shulman KI, Reynolds CF, 3rd. Pharmacotherapy of bipolar disorder in old age: review and recommendations. The American journal of geriatric psychiatry : official journal of the American Association for Geriatric Psychiatry. 2004;12(4):342-57. doi: 10.1176/appi.ajgp.12.4.342. PubMed PMID: 15249272.

132. Forester BP, Streeter CC, Berlow YA, Tian H, Wardrop M, Finn CT, Harper D, Renshaw PF, Moore CM. Brain lithium levels and effects on cognition and mood in geriatric bipolar disorder: a lithium-7 magnetic resonance spectroscopy study. The American journal of geriatric psychiatry : official journal of the American Association for Geriatric Psychiatry. 2009;17(1):13-23. Epub 2008/07/16. doi: JGP.0b013e318172b3d0 [pii] 10.1097/JGP.0b013e318172b3d0. PubMed PMID: 18626002.
133. Feng Y, Pollock BG, Ferrell RE, Kimak MA, Reynolds CF, 3rd, Bies RR. Paroxetine: population pharmacokinetic analysis in late-life depression using sparse concentration sampling. Br J Clin Pharmacol. 2006;61(5):558-69. doi: 10.1111/j.1365-2125.2006.02629.x. PubMed PMID: 16669849; PubMed Central PMCID: PMC1885048.
134. Schulz KF, Altman DG, Moher D, Group C. CONSORT 2010 statement: updated guidelines for reporting parallel group randomized trials. Ann Intern Med. 2010;152(11):726-32. doi: 10.7326/0003-4819-152-11-201006010-00232. PubMed PMID: 20335313.
135. Moher D, Hopewell S, Schulz KF, Montori V, Gotzsche PC, Devereaux PJ, Elbourne D, Egger M, Altman DG. CONSORT 2010 explanation and elaboration: updated guidelines for reporting parallel group randomised trials. BMJ. 2010;340:c869. doi: 10.1136/bmj.c869. PubMed PMID: 20332511; PubMed Central PMCID: PMC2844943.
136. Tian L, Alizadeh AA, Gentles AJ, Tibshirani R. A simple method for detecting interactions between treatment and a large number of covariates 2012. Available from: http://www-stat.stanford.edu/~tibs/ftp/interactionpaper.pdf.
137. Krishnan A, Williams LJ, McIntosh AR, Abdi H. Partial Least Squares (PLS) methods for neuroimaging: a tutorial and review. NeuroImage. 2011;56(2):455-75. doi: 10.1016/j.neuroimage.2010.07.034. PubMed PMID: 20656037.
138. Vallesi A, McIntosh AR, Alexander MP, Stuss DT. FMRI evidence of a functional network setting the criteria for withholding a response. NeuroImage. 2009;45(2):537-48. doi: 10.1016/j.neuroimage.2008.12.032. PubMed PMID: 19162201.
139. Caplan JB, McIntosh AR, De Rosa E. Two distinct functional networks for successful resolution of proactive interference. Cerebral cortex. 2007;17(7):1650-63. doi: 10.1093/cercor/bhl076. PubMed PMID: 16968868.
140. Efron B, Tibshirani R. Bootstrap methods for standard errors: confidence intervals and other measures of statistical accuracy. Statist Sci. 1986;1(54-77).
