# Supplementary material for: Low-Dose Lithium for Mild Cognitive Impairment: A Pilot Randomized Clinical Trial
Source: JAMA Neurol. 2026 Mar 2;83(4):310–9. doi: 10.1001/jamaneurol.2026.0072 (PMC12954601; doi:10.1001/jamaneurol.2026.0072)
Supplement: Supplement 2. — eTable 1. Study Assessment Schedule and Instruments eTable 2. Serious Adverse Events by Treatment Group eTable 3. Summary of Adverse Events by Organ System and Treatment Group eTable 4. Individual Participant Changes from Baseline to 2 Years (Completers Analysis) eTable 5. Individual Participant Changes From Baseline to 2 Years in Aβ+ Participants (Completers Analysis) eTable 6. Individual Participant Changes from Baseline to 2 Years in Aβ− Participants (Completers Analysis) [file jamaneurol-e260072-s002.pdf]

Supplementary Online Content

Gildengers AG, Ibrahim TS, Anderson SJ, et al. Low-dose lithium for mild cognitive impairment: a pilot randomized clinical trial. *JAMA Neurol*. Published March 2, 2026. doi:10.1001/jamaneurol.2026.0072

eTable 1. Study Assessment Schedule and Instruments
eTable 2. Serious Adverse Events by Treatment Group
eTable 3. Summary of Adverse Events by Organ System and Treatment Group
eTable 4. Individual Participant Changes from Baseline to 2 Years (Completers Analysis)
eTable 5. Individual Participant Changes from Baseline to 2 Years in Aβ+ Participants (Completers Analysis)
eTable 6. Individual Participant Changes from Baseline to 2 Years in Aβ− Participants (Completers Analysis)

This supplemental material has been provided by the authors to give readers additional information about their work.

© 2026 Gildengers AG et al. JAMA Neurology.

eTable 1. Study Assessment Schedule and Instruments

**Assessment**	**Screening (T0)**		**RCT (T1, T2, T3)**			
	Initial	Comprehensive	Pre-RCT (T1)	Quarterly	1-y (T2)	2-y (T3)
Inclusion/Exclusion	X					
MINI	X					
**Cognitive**						
mTICS or Qmci	X				X	X
3MS (in person only)	X				X	X
HSCT or Trails A and B	X					
BVMT-R			X		X	X
CDR		X			X	X
CVLT-II			X		X	X
D-KEFS		X			X	X
E-Cog		X			X	X
RBANS		X			X	X
Clock		X			X	X
Digit Span		X			X	X
Boston Naming Test		X			X	X
NIH Toolbox			X		X	X
WRAT-4 Reading		X				
**Clinical**						
BARS (administered monthly)				X	X	X
CIRS-G		X			X	X
FSRP		X			X	X
Medication List		X			X	X
PASE		X		X	X	X
PHQ-9		X		X	X	X

© 2026 Gildengers AG et al. JAMA Neurology.

UKU		X		X	X	X
SRSE		X		X	X	X
PASS items	X				X	X
**Imaging**						
7T MRI			X		X	X
PET (Aβ)			X			X
**Laboratory**						
Blood-based biomarkers (biannual)			X		X	X
Safety Labs		X		X	X	X

Abbreviations: BARS, Brief Adherence Scale; BVMT-R, Brief Visual Memory Test-Revised; CDR, Clinical Dementia Rating; CIRS-G, Cumulative Illness Rating Scale-Geriatric; CVLT-II, California Verbal Learning Test-II; D-KEFS, Delis-Kaplan Executive Function System; E-Cog, Everyday Cognition scale; FSRP, Framingham Stroke Risk Profile; HSCT, Hayling Sentence Completion Test; MINI, Mini-International Neuropsychiatric Interview; mTICS, Modified Telephone Interview for Cognitive Status; PASE, Physical Activity Scale for the Elderly; PASS, Performance Assessment of Self-care Skills; PHQ-9, Patient Health Questionnaire-9 item; Qmci, Quick Mild Cognitive Impairment; RBANS, Repeatable Battery for the Assessment of Neuropsychological Status; SRSE, spontaneous reporting of adverse effects; UKU, Udvalg for Kliniske Undersøgelser Side Effect Rating Scale; WRAT-4, Wide Range Achievement Test-4; 3MS, Modified Mini-Mental Status.

© 2026 Gildengers AG et al. JAMA Neurology.

eTable 2. Serious Adverse Events by Treatment Group

System Organ Class/Preferred Term	Lithium (n = 41)		Placebo (n = 39)	
	Events	Participants, No. (%)	Events	Participants, No. (%)
**Blood and lymphatic system disorders**				
Leukopenia	1	1 (2.4)	0	0
**Cardiac disorders**				
Shortness of breath, pedal edema, pulmonary embolism, acute atrial flutter with RVR on chronic CHF	1	1 (2.4)	0	0
Congestive heart failure	1	1 (2.4)	0	0
Recurrence of atrial flutter	1	1 (2.4)	0	0
Shortness of breath	1	1 (2.4)	0	0
**Gastrointestinal disorders**				
Bleeding stomach ulcer	2	1 (2.4)	0	0
Nausea and left arm pain	0	0	1	1 (2.6)
**Infections and infestations**				
Community acquired pneumonia	2	2 (4.9)	0	0
Fever and shortness of breath	1	1 (2.4)	0	0
Infectious aortitis	1	1 (2.4)	0	0
COVID-19	0	0	1	1 (2.6)
**Injury, poisoning and procedural complications**				
Motor vehicle accident	1	1 (2.4)	0	0
Fall	1	1 (2.4)	1	1 (2.6)
Leg wounds	1	1 (2.4)	0	0
Etadolac-related ischemic colitis	1	1 (2.4)	0	0
Frequent falls	0	0	1	1 (2.6)
**Musculoskeletal and connective tissue disorders**				
Fall, fracture of right hip	1	1 (2.4)	0	0
Dislocation of right hip	2	1 (2.4)	0	0
Leg pain and swelling	1	1 (2.4)	0	0
Total knee arthroscopy	1	1 (2.4)	1	1 (2.6)
Bilateral hip and leg pain	0	0	1	1 (2.6)
**Neoplasms benign, malignant and unspecified (including cysts and polyps)**				
Radiation treatment of malignant skin neoplasm and osteomyelitis	1	1 (2.4)	0	0

© 2026 Gildengers AG et al. JAMA Neurology.

Lung cancer	0	0	1	1 (2.6)
Prostate cancer	0	0	1	1 (2.6)
**Nervous system disorders**				
Subdural hemorrhage	0	0	2	1 (2.6)
Stroke	0	0	1	1 (2.6)
**Renal and urinary disorders**				
Acute kidney injury	2	1 (2.4)	0	0
**Respiratory, thoracic and mediastinal disorders**				
Rheumatoid lung disease[a]	0	0	2	1 (2.6)
Acute hypoxemia	0	0	1	1 (2.6)
Confusion and shortness of breath	0	0	1	1 (2.6)
**Surgical and medical procedures**				
Neck surgery	1	1 (2.4)	0	0
Total knee replacement	0	0	1	1 (2.6)
**Vascular disorders**				
Vasovagal episode	1	1 (2.4)	0	0
**Total serious adverse events**	**25**	**12 (29.3)**	**16**	**9 (23.1)**

Abbreviations: CHF, congestive heart failure; RVR, rapid ventricular response.
[a]One participant in the placebo group died from respiratory failure due to rheumatoid interstitial lung disease. Data are number of events and number (%) of participants affected. All events listed met criteria for serious adverse events requiring hospitalization, life-threatening events, or significant disability. Events are grouped by organ system in descending order of total frequency. Individual participants may have experienced multiple serious adverse events. Assessment of causality relationship to study medication was done for all serious adverse events. No serious adverse events were considered definitely related to study medication.

© 2026 Gildengers AG et al. JAMA Neurology.

eTable 3. Summary of Adverse Events by Organ System and Treatment Group

System Organ Class/Preferred Term	Lithium (n = 41)		Placebo (n = 39)	
	Events	Participants, No. (%)	Events	Participants, No. (%)
Total		41 (100)		37 (94.9)
**Blood and lymphatic system disorders**				
Increased calcium value	7	5 (12.2)	0	0
Pedal edema	2	1 (2.4)	3	1 (2.6)
Electrolyte abnormality	0	0	1	1 (2.6)
**Cardiac disorders**				
Congestive heart failure	2	1 (2.4)	0	0
**Endocrine disorders**				
Increase TSH value	7	7 (17.1)	5	3 (7.7)
**Eye disorders**				
Accommodation disturbance	1	1 (2.4)	0	0
Blurred vision	2	1 (2.4)	0	0
**Gastrointestinal disorders**				
Diarrhea	21	12 (29.3)	11	6 (15.4)
Nausea	6	4 (9.8)	3	2 (5.1)
Stomach discomfort	5	4 (9.8)	1	1 (2.6)
Constipation	8	4 (9.8)	4	3 (7.7)
Stomach pain	3	1 (2.4)	1	1 (2.6)
Indigestion	2	1 (2.4)	3	2 (5.1)
Vomiting	1	1 (2.4)	0	0
Gastrointestinal pain	0	0	1	1 (2.6)
Stomach ache	0	0	1	1 (2.6)
**General disorders and administration site conditions**				
Tiredness	19	12 (29.3)	9	6 (15.4)
Sleepiness	19	10 (24.4)	12	9 (23.1)
Headache	11	5 (12.2)	2	2 (5.1)
Dizziness	10	5 (12.2)	9	6 (15.4)
Unsteadiness	8	5 (12.2)	7	3 (7.7)
Dry mouth	7	4 (9.8)	6	3 (7.7)
Fatigue	4	4 (9.8)	4	3 (7.7)
Concentration difficulties	3	3 (7.3)	2	2 (5.1)
Insomnia	2	2 (4.9)	10	6 (15.4)
Fever	2	2 (4.9)	0	0
Facial pain	2	1 (2.4)	0	0

© 2026 Gildengers AG et al. JAMA Neurology.

Hot flashes	2	1 (2.4)	0	0
Itchiness	2	1 (2.4)	2	1 (2.6)
Metallic taste	1	1 (2.4)	0	0
Disorientation	1	1 (2.4)	0	0
Night sweats	1	1 (2.4)	1	1 (2.6)
Palpitations	1	1 (2.4)	1	1 (2.6)
Weight loss	1	1 (2.4)	1	1 (2.6)
Flu-like symptoms	0	0	1	1 (2.6)
Hives	0	0	2	1 (2.6)
Hungriness	0	0	1	1 (2.6)
Inner unrest	0	0	1	1 (2.6)
Leg cramps	0	0	1	1 (2.6)
Somniloquy	0	0	1	1 (2.6)
**Infections and infestations**				
Sinusitis	2	1 (2.4)	0	0
Lyme disease	1	1 (2.4)	0	0
**Injury, poisoning and procedural complications**				
Fall	2	2 (4.9)	1	1 (2.6)
Falls, recurrent	1	1 (2.4)	0	0
Mechanical fall	1	1 (2.4)	0	0
**Metabolism and nutrition disorders**				
Weight gain	9	7 (17.1)	5	4 (10.3)
Increased thirst	2	2 (4.9)	1	1 (2.6)
**Musculoskeletal and connective tissue disorders**				
Body aches	2	1 (2.4)	0	0
Heaviness in limbs	2	1 (2.4)	0	0
Heel pain	1	1 (2.4)	0	0
Lower back pain	1	1 (2.4)	0	0
Muscle aches	0	0	1	1 (2.6)
Muscle cramps	0	0	1	1 (2.6)
Muscle fatigue	0	0	1	1 (2.6)
**Nervous system disorders**				
Tremor	13	10 (24.4)	18	6 (15.4)
Facial nerve palsy	2	1 (2.4)	0	0
Memory difficulties	2	2 (4.9)	1	1 (2.6)
Hypnogogic hallucination	1	1 (2.4)	0	0
Akathisia	1	1 (2.4)	0	0
**Psychiatric disorders**				

© 2026 Gildengers AG et al. JAMA Neurology.

Irritability	3	3 (7.3)	1	1 (2.6)
Stuttering	2	1 (2.4)	0	0
Depression	1	1 (2.4)	0	0
Increased dream activity	1	1 (2.4)	3	2 (5.1)
Increased sleep duration	1	1 (2.4)	0	0
Reduced emotional flattening	0	0	2	1 (2.6)
Twitching in sleep	0	0	1	1 (2.6)
**Renal and urinary disorders**				
Increased creatinine	16	12 (29.3)	21	12 (30.8)
Increased urinary frequency	9	4 (9.8)	14	6 (15.4)
Micturation difficulty	1	1 (2.4)	0	0
**Reproductive system and breast disorders**				
Erectile dysfunction	0	0	2	1 (2.6)
**Respiratory, thoracic and mediastinal disorders**				
Cough	3	1 (2.4)	0	0
Shortness of breath	2	2 (4.9)	0	0
Sinus congestion	2	1 (2.4)	0	0
Lung inflammation	0	0	2	1 (2.6)
**Skin and subcutaneous tissue disorders**				
Hair loss	4	2 (4.9)	6	4 (10.3)
Rash	1	1 (2.4)	0	0
Mouth ulcers	0	0	2	1 (2.6)
**Vascular disorders**				
Edema	11	5 (12.2)	1	1 (2.6)

Abbreviation: TSH, thyroid-stimulating hormone.
All adverse events were classified as other (nonserious) events. Percentages calculated on the basis of total number of participants in each treatment group. Events reported if occurring in 1 or more participants in either treatment group. Participants may have experienced multiple events within the same category.

© 2026 Gildengers AG et al. JAMA Neurology.

eTable 4. Individual Participant Changes from Baseline to 2 Years (Completers Analysis)

Variable	Lithium			Placebo			Difference (95% CI)	P Value[a]
	n	Mean Change (T1 to T3)	SD	n	Mean Change (T1 to T3)	SD		
PACC score	37	−0.83	3.62	28	−0.958	2.01	0.13 (−1.29, 1.54)	.86[b]
BVMT-R	36	−0.69	2.72	31	−0.452	2.77	−0.24 (−1.58, 1.11)	.72
CVLT-II	37	−1.51	3.17	31	−3.000	2.25	1.49 (0.17, 2.81)	.04[c]
BDNF (log-transformed)	33	−0.10	1.21	29	0.068	1.42	−0.17 (−0.84, 0.51)	.58
Cortical volume, mm^3	28	−6345.6	11 137.3	21	−5134.0	6855.9	−1211.6 (−6411.0, 3987.8)	.64[b]
Hippocampal volume, mm^3	28	−158.3	217.1	21	−268.5	266.4	110.2 (−33.9, 254.3)	.12
Hippocampal volume change, %	28	−2.24	3.08	21	−4.017	4.16	1.78 (−0.41, 3.97)	.09

Abbreviations: BDNF, brain-derived neurotrophic factor; BVMT-R, Brief Visuospatial Memory Test-Revised; CI, confidence interval; CVLT-II, California Verbal Learning Test-II; PACC, Preclinical Alzheimer Cognitive Composite; T1, baseline; T3, end of study.
Difference = Lithium − Placebo. Positive values indicate less decline (better outcome) in the lithium group. Confidence intervals calculated using the same statistical test as the corresponding P value.
[a]P values calculated using standard 2-sample t test unless indicated otherwise.
[b]P value calculated using Satterthwaite t test.
[c]Nominally significant at $\alpha = .05$ but does not survive correction for multiple comparisons (significance threshold: $P < .01$).

© 2026 Gildengers AG et al. JAMA Neurology.

eTable 5. Individual Participant Changes from Baseline to 2 Years in Aβ+ Participants (Completers Analysis)

Variable	Lithium			Placebo			Difference (95% CI)	P Value[a]
	n	Mean Change (T1 to T3)	SD	n	Mean Change (T1 to T3)	SD		
PACC score	9	−3.58	6.13	9	−2.958	1.58	−0.62 (−5.39, 4.15)	.77[b]
BVMT-R	10	−2.10	2.73	9	−1.667	2.92	−0.43 (−3.18, 2.32)	.74
CVLT-II	10	−1.70	4.03	9	−4.333	2.45	2.63 (−0.59, 5.86)	.11
BDNF (log-transformed)	10	−0.02	0.95	8	0.615	1.17	−0.64 (−1.74, 0.47)	.22
Cortical volume, mm^3	7	−12 656.7	11 101.3	8	−10 511.1	5611.5	−2145.6 (−12 717.2, 8426.0)	.64
Hippocampal volume, mm^3	7	−265.8	201.4	8	−475.7	268.0	209.9 (−53.2, 473.0)	.11
Hippocampal volume change, %	7	−3.83	3.13	8	−7.22	4.55	3.39 (−0.95, 7.73)	.12

Abbreviations: Aβ+, amyloid beta positive; BDNF, brain-derived neurotrophic factor; BVMT-R, Brief Visuospatial Memory Test-Revised; CI, confidence interval; CVLT-II, California Verbal Learning Test-II; PACC, Preclinical Alzheimer Cognitive Composite; T1, baseline; T3, end of study.

Difference = Lithium − Placebo. Positive values indicate less decline (better outcome) in the lithium group. Confidence intervals calculated using the same statistical test as the corresponding P value.

[a]P values calculated using standard 2-sample t test unless indicated otherwise.

[b]P value calculated using Satterthwaite t test.

© 2026 Gildengers AG et al. JAMA Neurology.

eTable 6. Individual Participant Changes from Baseline to 2 Years in Aβ− Participants (Completers Analysis)

Variable	Lithium			Placebo			Difference (95% CI)	P Value[a]
	n	Mean Change (T1 to T3)	SD	n	Mean Change (T1 to T3)	SD		
PACC score	25	0.09	1.79	18	−0.072	1.42	0.16 (−0.83, 1.15)	.76
BVMT-R	23	−0.04	2.65	20	−0.20	2.61	0.16 (−1.46, 1.78)	.85
CVLT-II	24	−1.75	2.80	20	−2.60	2.30	0.85 (−0.70, 2.40)	.28
BDNF (log-transformed)	21	−0.22	1.03	20	−0.15	1.51	−0.07 (−0.89, 0.75)	.87
Cortical volume, mm^3	21	−4244.6	10 578.6	13	−1824.9	5369.6	−2419.7 (−8021.6, 3182.2)	.39[b]
Hippocampal volume, mm^3	21	−122.5	214.6	13	−141.0	173.6	18.5 (−118.8, 155.8)	.79
Hippocampal volume change, %	21	−1.71	2.95	13	−2.05	2.40	0.34 (−1.55, 2.23)	.73

Abbreviations: Aβ−, amyloid beta negative; BDNF, brain-derived neurotrophic factor; BVMT-R, Brief Visuospatial Memory Test-Revised; CI, confidence interval; CVLT-II, California Verbal Learning Test-II; PACC, Preclinical Alzheimer Cognitive Composite; T1, baseline; T3, end of study.

Difference = Lithium − Placebo. Positive values indicate less decline (better outcome) in the lithium group. Confidence intervals calculated using the same statistical test as the corresponding P value.

[a]P values calculated using standard 2-sample t test unless indicated otherwise.

[b]P value calculated using Satterthwaite t test.

© 2026 Gildengers AG et al. JAMA Neurology.
