# Supplementary material for: Low-Dose Lithium for Mild Cognitive Impairment: A Pilot Randomized Clinical Trial
Source: JAMA Neurol. 2026 Mar 2;83(4):310–9. doi: 10.1001/jamaneurol.2026.0072 (PMC12954601; doi:10.1001/jamaneurol.2026.0072)
Supplement: Supplement 3. — Data Sharing Statement [file jamaneurol-e260072-s003.pdf]

Data Sharing Statement

Gildengers. Low-Dose Lithium for Mild Cognitive Impairment. *JAMA Neurol*. Published March 02, 2026. doi:10.1001/jamaneurol.2026.0072

Data
Additional Information: ClinicalTrials.gov Identifier: NCT03185208
Data available: Yes
Data types: Deidentified participant data
How to access data: Deidentified individual participant data that underlie the results reported in this article will be made available to qualified researchers through the National Centralized Repository for Alzheimer's Disease and Related Dementias (NCRAD; https://ncrad.iu.edu/). Data will be deposited within 12 months of publication. Access requires an approved data request through NCRAD's standard application process.
When available: beginning date: 12-31-2026

Supporting Documents
Document types: None

Additional Information
Who can access the data: Data will be made available to qualified researchers through the National Centralized Repository for Alzheimer's Disease and Related Dementias (NCRAD; https://ncrad.iu.edu/).
Types of analyses: Data will be made available pending approval through NCRAD's standard application process.
Mechanisms of data availability: Data will be made available approved through NCRAD's standard application process.
